# Quantum refinement with electron diffraction and X-ray free-electron laser data: comparative study of ribonucleotide reductase dimetal site

**DOI:** 10.1107/S1600576725011264

**Published:** 2026-02-09

**Authors:** Kristoffer J. M. Lundgren, Xiaoli Sun, Laura Pacoste, Rohit Kumar, Gerhard Hofer, Hongyi Xu, Xiaodong Zou, Martin Högbom, Esko Oksanen, Ulf Ryde

**Affiliations:** a Division of Computational Chemistry, Chemical Centre Lund University PO Box 124 SE-221-00Lund Sweden; b Department of Chemistry Stockholm University SE-106-91Stockholm Sweden; c Department of Biochemistry and Science for Life Laboratory Stockholm University SE-106-91Stockholm Sweden; d European Spallation Source PO Box 176 SE-221-00Lund Sweden; Oak Ridge National Laboratory, USA; North Carolina State University, USA

**Keywords:** quantum refinement, single-crystal X-ray crystallography, X-ray free-electron laser diffraction, microcrystal electron diffraction, ribonucleotide reductase R2a, binuclear Fe_2_ site, water, hydroxide, oxo group

## Abstract

Quantum refinement (QR) is an approach in which the empirical restraints used in standard structural refinement to ensure that the details of the structure, *e.g.* bond lengths and angles, make chemical sense are replaced by more accurate quantum mechanical calculations for a small but interesting part of the structure. QR has previously been used for X-ray and neutron crystallography, cryogenic electron microscopy, nuclear magnetic resonance, and extended X-ray absorption fine structure. Here, QR is used for the first time for X-ray free-electron laser (XFEL) crystallography and microcrystal electron diffraction (MicroED). As a test case, we use six structures of the R2a protein of ribonucleotide reductase, concentrating on the binuclear Fe_2_ site in either the oxidized (Fe_2_^III^) or reduced (Fe_2_^II^) state, two each from single-crystal X-ray (SCX) crystallography, XFEL crystallography or MicroED. The results show that QR works well for data from all three radiation sources, even though scattering factors for neutral atoms had to be used for MicroED. QR corrects unrealistically short Fe—O distances in the reduced SCX structure and gives improved real-space *Z* scores for the reduced MicroED structure. The three methods give similar structures, apart from variation in the weak water ligands and in the binding of carboxylate groups (monodentate, bidentate or a mixture). By performing QR for three protonation states of the bridging solvent molecule, we could show that it is undoubtedly a water molecule in the reduced XFEL and MicroED structures (it is not present in the SCX structure) and that it is not water in the oxidized structures. The XFEL data indicate that it is O^2−^ in the oxidized XFEL structure, in agreement with the spectroscopic results. However, for the SCX structure, O^2−^ and OH^−^ give comparable results, whereas OH^−^ is slightly preferred in the MicroED structure. This indicates that the SCX and MicroED structures may be partly photoreduced during data collection.

## Abstract 2

For the first time quantum refinement has been performed with X-ray free-electron laser and microcrystal electron diffraction data for the dinuclear iron site in the R2a protein of ribonucleotide reductase. The method is shown to work well and can be used to determine the protonation state of the bridging solvent molecule and deduce photoreduction in two of the structures.

## 1. Introduction

Structural information at the atomic level is key to understanding the function of biological macromolecules. Traditionally, the vast majority of such information has been gained by single-crystal X-ray (SCX) crystallography. During recent years, cryogenic electron microscopy has become a competitive alternative. Neutron crystallography has long been used to locate hydrogen atoms, but due to experimental challenges only a few hundred protein structures are available so far. The same applies to electron crystallography (Unwin & Henderson, 1975 ▸; Henderson *et al.*, 1990 ▸), although in recent years new technical solutions have renewed interest in this technique (Saha *et al.*, 2022 ▸). Whereas X-rays are scattered by electrons, neutrons are scattered by nuclei and electrons by the electrostatic potential. Therefore, the various techniques may provide different types of complementary information about the structure, particularly for hydrogen atoms (Marques *et al.*, 2019 ▸).

Common to most of the above-mentioned methods is that the experimental information available for low- and medium-resolution macromolecular structures is not enough to determine the exact positions of all atoms. Therefore, the structure determination is supplemented by empirical data, such as information on ideal bond lengths, angles, dihedral angles *etc.* (Engh & Huber, 1991 ▸; Engh & Huber, 2012 ▸; Moriarty *et al.*, 2016 ▸). In terms of computational chemistry, this is a molecular mechanics (MM) force field. Therefore, standard crystallographic refinement employs a target function of the type 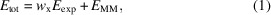
where *E*_exp_ is the experimental target function, describing how well the current model reproduces the experimental raw data, and *E*_MM_ represents the empirical restraints. The weight factor *w*_x_ determines the relative importance of the two terms.

The empirical restraints are typically obtained from small-molecule crystallographic structures or as averages over a large number of high-resolution macromolecular structures. Therefore, they are accurate for amino acids and nucleic acids, as well as common biological cofactors. However, for substrates, inhibitors and ligands, less information is available and the restraints may be less accurate. For structures with unusual chemical bonding (*e.g.* reaction intermediates) and metal sites, it may even be hard to set up reasonable empirical restraints, because the structure depends on the oxidation and spin state of the metal, as well as on the nature of all the ligands (Hu & Ryde, 2011 ▸). These problems can be solved by combining crystallographic refinement with quantum mechanical (QM) methods for a small but interesting part of the structure (called the QM region in the following) (Ryde *et al.*, 2002 ▸). This approach is called quantum refinement (QR) (Bergmann *et al.*, 2022 ▸) and employs an energy function of the form
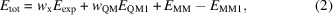
where *E*_QM1_ is the QM energy of the QM region and *E*_MM1_ is the MM energy of the QM region. *w*_QM_ is another empirical scaling factor, which is needed because the empirical restraints are normally of a statistical nature whereas *E*_QM1_ is a physical energy.

QR was originally developed for X-ray crystallography (Ryde *et al.*, 2002 ▸), but it was later extended to NMR structure refinement (Hsiao *et al.*, 2005 ▸), X-ray absorption fine-structure (EXAFS) measurements (Hsiao *et al.*, 2006 ▸), neutron crystallography (Caldararu *et al.*, 2019 ▸) and cryogenic electron microscopy (Lundgren *et al.*, 2024 ▸). We have shown that with QR we can locally improve crystal structures (Ryde & Nilsson, 2003 ▸), determine the protonation states of active-site residues (Nilsson & Ryde, 2004 ▸; Cao *et al.*, 2017 ▸; Bergmann *et al.*, 2021*c* ▸), determine the oxidation states of metals (Rulíšek & Ryde, 2006 ▸), detect photoreduction of metal sites (Rulíšek & Ryde, 2006 ▸; Söderhjelm & Ryde, 2006 ▸) and determine what atoms are actually seen in crystal structures (Cao *et al.*, 2020 ▸; Bergmann *et al.*, 2021*b* ▸). Several other groups have implemented similar approaches (Bergmann *et al.*, 2022 ▸; Hsiao *et al.*, 2010 ▸; Fadel *et al.*, 2015 ▸; Yan *et al.*, 2021 ▸; Yan *et al.*, 2024 ▸).

In this study, we continue this development by extending QR to structures obtained by X-ray free-electron laser (XFEL) serial crystallography and microcrystal electron diffraction (MicroED). XFEL data are in principle comparable to SCX but are obtained from ultrabright femtosecond pulses in a diffraction-before-destruction regime, minimizing radiation damage and thereby avoiding structural changes due to photoreduction at metal sites (Liu & Lee, 2019 ▸). Consequently, standard refinement protocols, and by extension QR, can be used without modification, and with no expected impact of photoreduction on the structure.

MicroED requires different considerations. Since electrons are scattered by the electrostatic potential rather than electron density, different atomic scattering factors should be used. Typically, cryo-EM and MicroED data are refined with atomic scattering factors for neutral, isolated and spherically symmetric atoms, as tabulated in *International Tables for Crystallography* (Colliex *et al.*, 2006 ▸). The electron scattering table for neutral atoms with Gaussian parameterization is implemented in *phenix.refine* (Liebschner *et al.*, 2019 ▸). Potential complications with MicroED data include photoreduction at the metal site (Hattne *et al.*, 2018 ▸), multiple scattering, and slightly reduced completeness from the missing wedge when crystals exhibit preferred orientation. The key question is whether the data quality is sufficient for QR to yield conclusions comparable to those from XFEL data.

As a test case, we use the structure of ribonucleotide reductase, for which XFEL and MicroED data are available in two different oxidation states. This enzyme converts ribo­nucleotides to deoxyribonucleotides by removing the 2′-OH group on the ribose moiety (Greene *et al.*, 2020 ▸). We study a class I ribonucleotide reductase from *Escherichia coli*. The active enzyme is a complex of two dimeric proteins, called R1a and R2a. R1a binds the substrate and allosteric effectors, whereas R2a contains a dimeric Fe site that is used to form a stable tyrosine radical, which is used in the catalysis in R1a. We study the R2a protein and concentrate the investigation on the oxidized (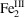, metR2a) and reduced (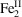, redR2a) active sites. We compare the results with data from standard X-ray crystallographic structures (Högbom *et al.*, 2003 ▸; Logan *et al.*, 1996 ▸) and investigate whether the three approaches give comparable results.

In the oxidized state, the Fe_2_ cluster contains a bridging solvent-derived ligand. Spectroscopic investigations indicate that it is a doubly deprotonated O^2−^ group (Sjöberg *et al.*, 1982 ▸; Bunker *et al.*, 1987 ▸; Scarrow *et al.*, 1986 ▸), stabilized by the charges of the two Fe^3+^ ions. In standard SCX or XFEL macromolecular studies, hydrogen atoms are not discerned and the protonation state of solvent molecules can therefore not be determined. However, we have previously shown that QR can settle protonation states, because different proton­ation states give different metal–O distances and also affect the metal–ligand distances of the other ligands (Nilsson & Ryde, 2004 ▸; Bergmann *et al.*, 2022 ▸). Here, we study whether the three approaches have similar discriminating power between possible protonation states of the solvent ligand in the metR2a structure, and we also determine the protonation state of a solvent molecule found in the XFEL and MicroED structures of redR2a.

## 2. Methods

We have applied QR to three structures of oxidized R2a (metR2a) and three structures of reduced R2a (redR2a), *viz.* two published X-ray structures of wild-type R2a (Högbom *et al.*, 2003 ▸; Logan *et al.*, 1996 ▸), two recent XFEL structures of the Tyr122Phe mutant of R2a (Hofer *et al.*, 2025 ▸) and two new MicroED structures of the same mutant. All structures come from *E. coli* R2a.

### 2.1. Crystallization for MicroED and grid preparation

Microcrystals of the Y122F mutant of metR2a were prepared using the RaMiC method (Hofer *et al.*, 2025 ▸). A seed stock was made by growing large metR2a crystals (space group *P*2_1_2_1_2_1_) via batch crystallization as follows. Two parts protein solution (55–60 mg ml^−1^ in 25 m*M* HEPES-Na pH 7.0, 50 m*M* NaCl) were mixed with one part crystallization buffer (25% PEG 3350, 0.1 *M* bis-Tris pH 5.5) and the resulting mixture was incubated for 24 h. Crystals were crushed in 5 µl of crystallization buffer, transferred to 45 µl of the same solution and then vortexed with steel beads to produce the seed stock, which was diluted tenfold. To generate microcrystals with the RaMiC procedure, 23.5 µl of seed stock was mixed with 20 µl of protein stock solution and vortexed in short intervals (5–10 s). Since the solution remained clear, 2 µl of 50% PEG was added and the mixture was vortexed again, forming a turbid suspension of microcrystals.

Grids were prepared by diluting the sample 50-fold and applying 1 µl onto a QUANTIFOIL holey carbon grid (CF-1.2/1.3, 300 mesh). Excess liquid was manually blotted from the back of the grid using filter paper before plunge freezing in liquid ethane.

Crystallization and transmission electron microscope (TEM) grid preparation of redR2a were performed under strict anaerobic conditions in a custom glove box, including plunge freezing in liquid ethane (Xu *et al.*, 2025 ▸). The full procedure of how the redR2a crystals were prepared has been reported in detail by Pacoste (2025 ▸). In brief, the protein was reduced by adding 50 m*M* sodium dithionite to the protein stock solution prior to crystallization. Crystallization followed the RaMiC procedure with the same crystallization conditions as for metR2a microcrystals, but with 2 m*M* sodium dithionite added to the crystallization buffer. Grids were prepared inside the glove box by diluting the slurry 50-fold and depositing 1 µl on the grid, followed by ∼10 s blotting of the back and plunge freezing in liquid ethane. The grids were transferred out of the glove box under liquid ethane and were then transferred to liquid nitrogen prior to clipping and transfer into the microscope.

### 2.2. MicroED data collection and processing

All MicroED datasets were collected on a Titan Krios cryo-TEM (ThermoFisher Scientific) operating at 300 kV with a Ceta-D CMOS detector. The software *EPU-D* (https://documents.thermofisher.com/TFS-Assets/MSD/Product-Updates/epu-d-release-notes-1-23.pdf) was used for MicroED data collection.

MetR2a MicroED data were collected under varying beam conditions to balance high-resolution signal acquisition and data completeness. Exact beam and data collection parameters for each dataset are provided in Table S1 in the supporting information. Several datasets were acquired at low flux (≤0.05 e^−^ Å^−2^ s^−1^) using exposure times of 0.5–1 s. This approach enabled the collection of datasets with extended angular coverage (up to 99.5°) while maintaining a low total fluence (1.0–2.5 e^−^ Å^−2^), minimizing radiation damage. To enhance resolution, a subset of datasets were collected at higher flux (0.21 e^−^ Å^−2^ s^−1^) over narrower tilt ranges (19.8°), resulting in an accumulated fluence of 10.2 e^−^ Å^−2^ per dataset. Three additional datasets were acquired with a wider tilt step (0.5°) and extended range (64.5–79.5°), yielding the highest accumulated fluences (13.3–16.4 e^−^ Å^−2^) among those included in the final merge.

The redR2a crystals diffracted more weakly on average than the metR2a microcrystals. To improve the high-resolution signal, all datasets were collected using a higher beam flux. Data were collected at a camera length of 1.35 m with identical beam settings: spot size 8, C2 aperture 20 µm and beam size 1.5 µm, yielding a flux of 0.19 e^−^ Å^−2^. Detailed parameters are provided in Table S2. A tilt increment of 1° per frame was used to extend the angular coverage. Datasets spanned up to 59° with an average total fluence of 8.3 e^−^ Å^−2^. Exposure times ranged from 1 to 1.5 s, resulting in individual dataset fluences of 3.8–15.8 e^−^ Å^−2^.

Data were converted from MRC to IMG format using an in-house script and data reduction was performed with *XDS* (Kabsch, 2010 ▸). Datasets were clustered, scaled and merged using *edtools* (Smeets *et al.*, 2021 ▸). For metR2a, the final cluster included 22 crystals with data to 2.00 Å resolution, 88.1% completeness, CC_1/2_ of 98.4% and *I*/σ(*I*) of 5.73 (Table 1 ▸). In the case of redR2a, the final merged dataset included 69 crystals with data to 2.20 Å resolution, completeness of 85.8%, CC_1/2_ of 97.8% and *I*/σ(*I*) of 10.22 (Table 1 ▸). The resolution cut-off for refinement was intentionally kept generous (2.00 Å for metR2a and 2.20 Å for redR2a). The reason for this is that, despite weak signal in the outer resolution shell (CC_1/2_ of 9.1% for metR2a and 13.8% for redR2a), these reflections contributed meaningfully to refinement, which is reflected in the *R*_free_ values of 38.1% (metR2a) and 32.5% (redR2a) in the outer resolution bin.

### 2.3. Quantum refinement

All QR calculations employed the *QRef* interface (Lundgren *et al.*, 2024 ▸), which is an interface between the crystallography software *Phenix* (Liebschner *et al.*, 2019 ▸) and the QM software *ORCA* (Neese *et al.*, 2020 ▸).

For the four published SCX and XFEL structures [with PDB IDs 1mxr (Högbom *et al.*, 2003 ▸), 1xik (Logan *et al.*, 1996 ▸), 9sig and 9sih (Hofer *et al.*, 2025 ▸)], coordinates, occupancies, atomic displacement parameters (ADPs) and structure factors were downloaded from the Protein Data Bank (Berman *et al.*, 2000 ▸), together with the space group, unit-cell parameters, resolution limits, *R* values and the test set used for the evaluation of the *R*_free_ value (this last was not available for 1xik). For the new MicroED structures, QR was started after a normal refinement without QM. The MicroED data were processed to merge Friedel pairs.

Protons were added to the QM region with *phenix.ready_set*. For all structures, reciprocal-space QR with *QRef* was performed using *phenix.refine*, involving three macrocycles of coordinate and individual isotropic ADP refinement. In the coordinate refinement, only coordinates of the QM region, as well as the part of the residues outside of the QM region for which a covalent bond was cut, were allowed to move. For the ADP refinement part of the macrocycles, the ADPs for the whole protein were allowed to change. The *w*_QM_ weight factor in equation (2) was set to 7.5 (kcal mol^−1^)^–1^, as previously recommended (Lundgren *et al.*, 2024 ▸).

The QM calculations were performed with the TPSS density-functional theory (DFT) method (Tao *et al.*, 2003 ▸) and the def2-SV(P) basis set (Schäfer *et al.*, 1992 ▸). We employed the DFT-D4 dispersion correction (Caldeweyher *et al.*, 2019 ▸) in all calculations. This level of theory has been successfully employed in many previous QR studies of various proteins (Cao *et al.*, 2017 ▸; Cao *et al.*, 2018 ▸; Caldararu *et al.*, 2018 ▸; Lundgren *et al.*, 2024 ▸). It typically reproduces metal–ligand distances to within 0.05 Å (Cao & Ryde, 2019 ▸). Structures with a hybrid DFT method and larger basis sets [B3LYP-D4/def2-TZVP (Becke, 1988 ▸; Lee *et al.*, 1988 ▸; Becke, 1993 ▸; Weigend & Ahlrichs, 2005 ▸)] or with a continuum solvent (Cammi *et al.*, 2000 ▸; Bergmann *et al.*, 2021*a* ▸) were tested for one structure and give the same average real-space difference-density *Z* score (RSZD score) and differences in the Fe–ligand distances of no more than 0.03 Å (0.01 Å on average; Table S5).

**Figure 1:**
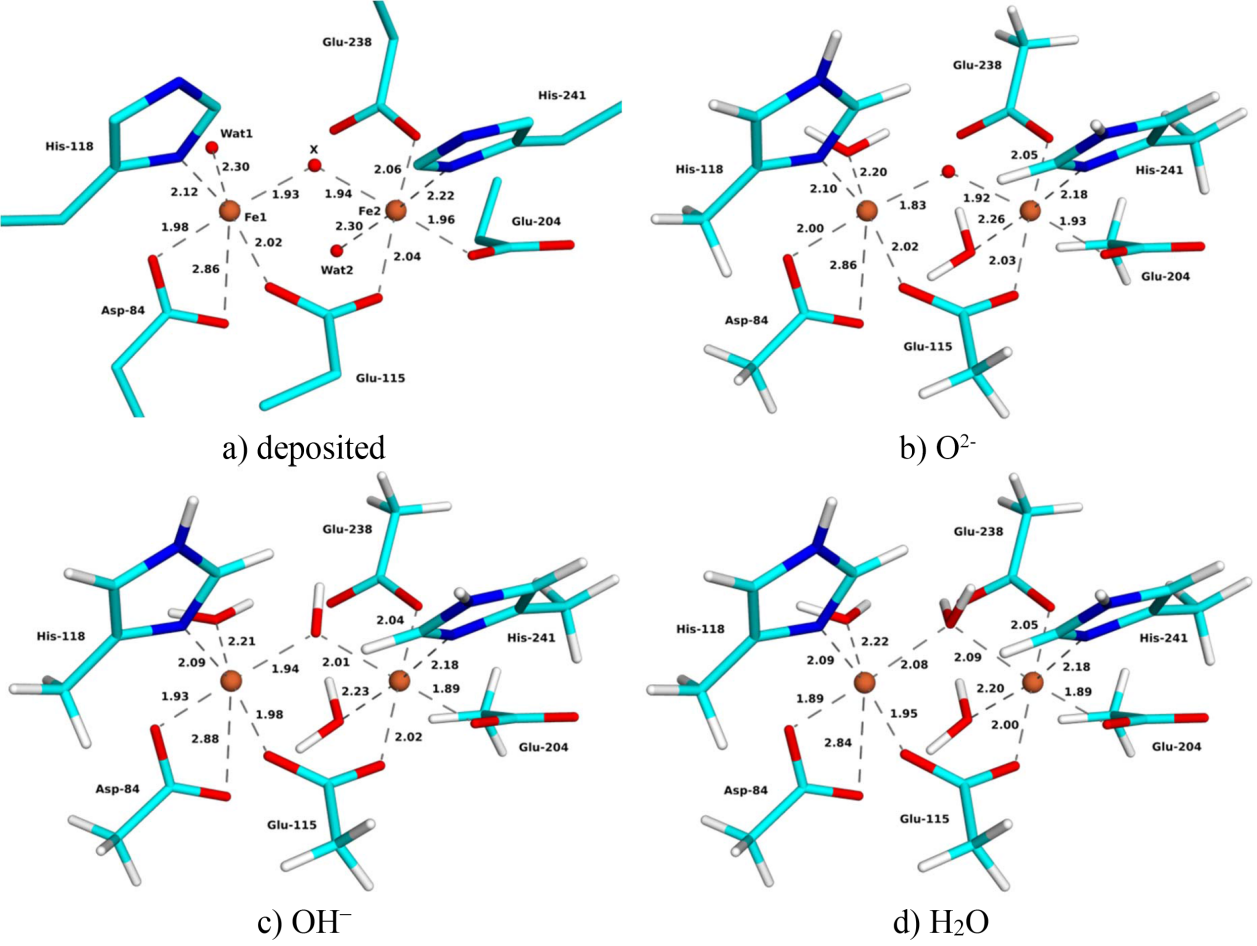
(*a*) Deposited and (*b*)–(*d*) QR SCX structures with (*b*) *X* = O^2−^, (*c*) *X* = OH^−^ and (*d*) *X* = H_2_O of oxidized metR2a. Key distances are given in ångströms.

The QM region included the two Fe ions in the binuclear site, as well as all first-sphere ligands: Asp-84, Glu-115, His-118, Glu-204, Glu-238, His-241 and 0–3 solvent molecules (three for the oxidized structures, one for the reduced MicroED and XFEL structures, and none for the reduced 1xik structure). All structures contain two polypeptide chains in the asymmetric unit. The QM calculations employed chain *A* in the SCX structures. For the two MicroED structures, chain *C* was used, because the first Fe atom in the structure belongs to this chain, but this chain also shows less flexibility than chain *B*. For the XFEL structures, we used chain *A* for metR2a but chain *B* for redR2a, because the metal site was unambiguous in chain *B*. To simplify the following discussion, the chain identifier is omitted. The QM region was truncated by hydrogen atoms (hydrogen link atoms), replacing the C*A* (Asp and His) or C*B* (Glu) atoms. Thus, the Asp and Glu ligands were modelled as acetate, whereas His was modelled as methylimidazole. Examples of the QM system are shown in Fig. 1 ▸.

The bridging solvent molecule was modelled as either O^2−^, OH^−^ or H_2_O, giving a net charge for the QM region of 0, +1 or +2 for the oxidized models and −2, −1 and 0 for the reduced models. The two Fe ions were either both Fe^III^ (oxidized state) or both Fe^II^ (reduced state). In either case, they were modelled in the high-spin state, but the eight or ten unpaired electrons were coupled antiferromagnetically to an open-shell singlet. It was treated by the broken-symmetry approach (Lovell *et al.*, 2001 ▸) using unrestricted Kohn–Sham formalism.

For simplicity, we will in the following call the Fe ion coordinating to His-118 Fe1 and the Fe ion coordinating to His-241 Fe2. Likewise, we call the water molecule coordinating only to Fe1 Wat1, the water coordinating only to Fe2 Wat2 (only oxidized structures) and the bridging solvent ligand *X*.

When comparing structures obtained with different *X* ligands, we use two quality measures. The real-space difference-density *Z* score, RSZD, shows how well each residue of the model explains the experimental data (Tickle, 2012 ▸). We have calculated RSZD scores for all residues in the QM region using the *edstats* software from the CCP4 suite (Tickle, 2012 ▸; Agirre *et al.*, 2023 ▸) and present the maximum absolute value for each residue. The strain energy is the difference in QM energy between the QR structure and a structure optimized with the same QM method without any experimental data (X-ray, XFEL or MicroED), but keeping the hydrogen link atoms fixed at the QR-structure coordinates (to keep the structure in the same local minimum as in the protein). It measures how much the QM region in the QR structure differs from an ideal QM structure.

For each structure, we first determined the ideal value of the *w*_x_ weight factor in equation (2) as has been described before (Figs. S1–S2 in the supporting information) (Lundgren *et al.*, 2024 ▸). The selected values (*w*_x_ = 1 for the SCX and MicroED structures and *w*_x_ = 3 for the XFEL structures) are similar to the values suggested by a standard *Phenix* refinement without QM (*w*_x_ = 1.1–2.1 for the SCX structures, 0.8–1.8 for the MicroED structures and 1.5–2.1 for the XFEL structures in the various macrocycles with the maximum-likelihood target function).

## 3. Results and discussion

In this investigation, we compare SCX, XFEL and MicroED structures of the oxidized and reduced states of R2a. All structures are evaluated with QR, using the *QRef* interface between *Phenix* and *ORCA* (Lundgren *et al.*, 2024 ▸). We focus our investigation on the binuclear Fe_2_ site and examine whether the bridging solvent molecule is best modelled as O^2−^, OH^−^ or H_2_O. The differences between these alternative models are so small that any global measures – such as *R* values – are not sensitive enough to discern any small local differences. Therefore, we used the RSZD score to compare the structures (Tickle, 2012 ▸). This essentially indicates the maximum absolute difference density for each residue considered and therefore should be as small as possible for a good structure and preferably not above 3. The results for the six structures are discussed in separate sections below.

### 3.1. Oxidized SCX structure

The 1mxr SCX structure from 2002 is at 1.42 Å resolution. It contains an Fe_2_ site in the oxidized 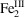 state. Each metal is approximately six-coordinate with carboxylate, His and water ligands. A solvent molecule and Glu-115 bridge the two Fe ions [Fig. 1 ▸(*a*)]. Asp-84 is halfway between mono- and bidentate binding, with Fe—OE2 and Fe—OE1 distances of 1.98–2.06 and 2.86–2.87 Å in the two subunits, respectively (Table 2 ▸). The Fe—O distances to the three (monodentate) Glu ligands are 1.96–2.07 Å, the Fe—N distances to the two His ligands are 2.11–2.22 Å and the Fe—O_Wat_ distances to the two water molecules (Wat1 and Wat2) are 2.22–2.30 Å. Wat2 is also rather close to Fe1, ∼3.2 Å. The bridging solvent mol­ecule (*X*) has Fe—O_*X*_ distances of 1.86–1.95 Å. The Fe—Fe distance is 3.35–3.38 Å.

The three QR structures (with *X* = O^2−^, OH^−^ or H_2_O) are quite similar to the deposited structure and to each other (Fig. 1 ▸). However, the Fe—O_*X*_ distances shorten as the charge of *X* becomes more negative (shown in Table 2 ▸): 2.08–2.09 Å for *X* = H_2_O, 1.94–2.01 Å for OH^−^ and 1.83–1.92 Å for O^2−^. As a consequence, most Fe—O/N distances to the other ligands elongate slightly (the only exception is Fe1—O_Wat1_ and to a minimal extent also Fe2—O_Glu238_). The Fe—Fe distance increases slightly in the QR structures, 3.37, 3.40 and 3.42 Å for O^2−^, OH^−^ and H_2_O, respectively.

The 16 distances in Table 2 ▸ are slightly closer to the original SCX structure for *X* = O^2−^ (average deviation of 0.03 Å) than for *X* = OH^−^ or H_2_O (average deviations of 0.04 and 0.07 Å, respectively). The strain energy is much higher for the QR structure with *X* = H_2_O (249 kJ mol^−1^) than for the other two structures (88 kJ mol^−1^ for OH^−^ and 89 kJ mol^−1^ for O^2−^). Likewise, the average RSZD score of the 11 residues in the QM system is higher with H_2_O (3.5) than with OH^−^ or O^2−^ (2.7; Table 2 ▸). The O^2−^ ligand gives better RSZD scores for four of the ligands, including the bridging solvent molecule, whereas OH^−^ gives better scores for two of the ligands and for the two Fe ions (Table S3). The maximum RSZD score of the 11 residues is lower for OH^−^ (4.3) than for O^2−^ (7.1) (in both cases for Fe1). The average difference of the 11 Fe—O/N coordinating distances in Table 2 ▸ between the QR structure and the corresponding vacuum-optimized QM structure is also higher for H_2_O (0.07 Å) than for the other two structures (0.03–0.04 Å).

Thus, QR unambiguously shows that the bridging ligand is not water, but it has problems discerning between O^2−^ and OH^−^. This may be because the differences in the structure caused by O^2−^ and OH^−^ are small. However, it may also indicate that the structure is photoreduced, so that it does not show a pure oxidation state. In fact, the Fe—O distances of the *X* ligand in the deposited structure, 1.83–1.96 Å (average 1.92 Å over the two distances in the two subunits), lie between the Fe—O distances obtained in the QR structures with O^2−^ (1.83 and 1.92 Å, average 1.88 Å) and OH^−^ (1.94 and 2.01 Å, average 1.97 Å). EXAFS and resonance Raman measurements have settled that the bridging solvent molecule is an O^2−^ bridge with Fe—O distances of 1.75–1.81 Å (Sjöberg *et al.*, 1982 ▸; Bunker *et al.*, 1987 ▸; Scarrow *et al.*, 1986 ▸).

The deposited 1mxr structure gives a slightly lower average RSZD score (2.3) than the QR structures with O^2−^ or OH^−^ (2.7) and the scores are better for most of the ligands, but they are worse for Fe1. This reflects the fact that the original structure was obtained without restraints on the metal ions and its ligands. It may also indicate that the structure is dynamic (the carboxylate groups can easily change between mono- and bidentate binding with essentially no barrier) and possibly photoreduced. The strain energy is much higher for the deposited structure (179–183 kJ mol^−1^) than for the QR structures (88–89 kJ mol^−1^), showing that QR improves the structure of the metal site.

### 3.2. Oxidized XFEL structure

**Figure 2:**
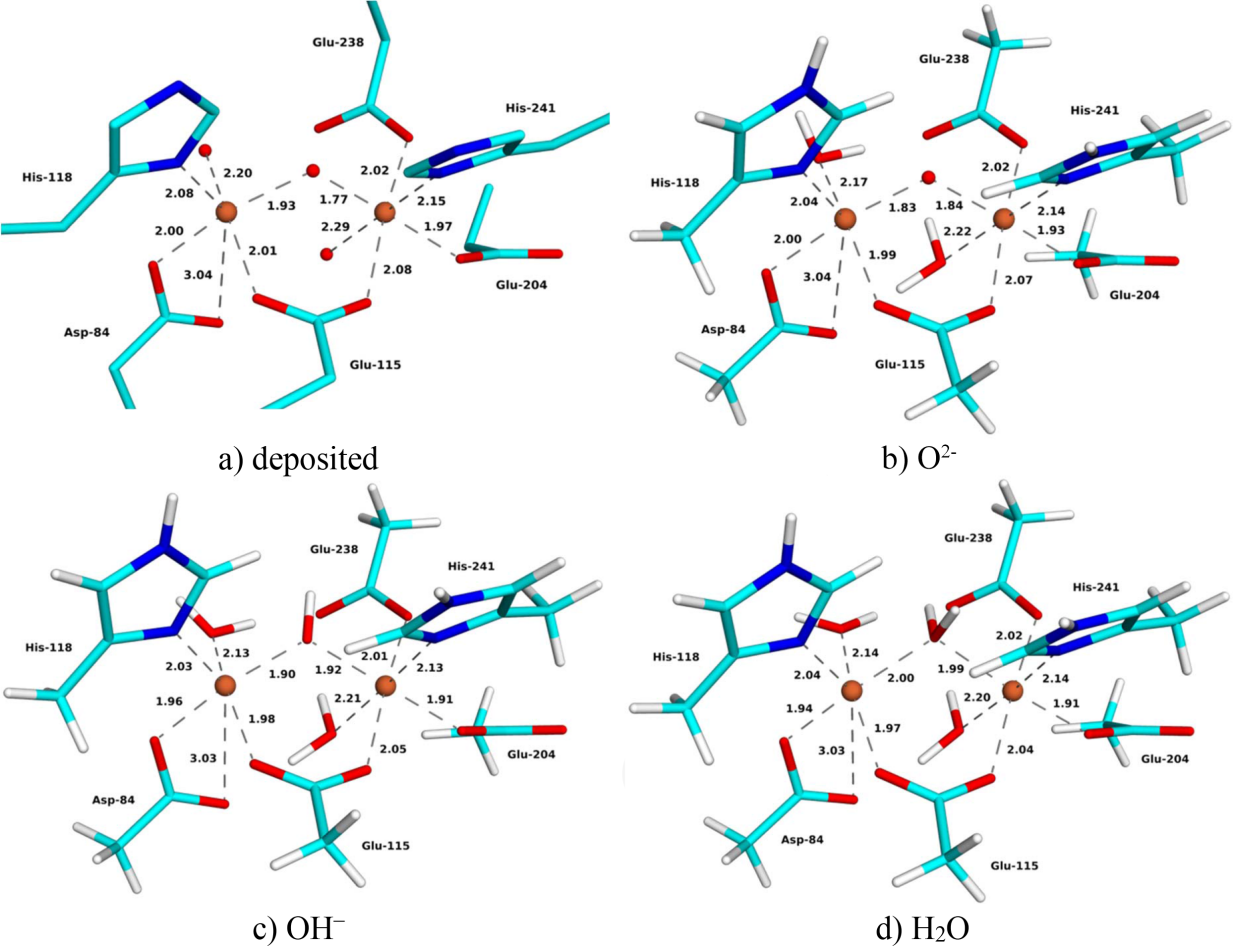
(*a*) Deposited and (*b*)–(*d*) QR XFEL structures with (*b*) *X* = O^2−^, (*c*) *X* = OH^−^ and (*d*) *X* = H_2_O of oxidized metR2a. Key distances are given in ångströms.

The deposited XFEL structure of oxidized metR2a (9sig) is at 1.9 Å resolution (Hofer *et al.*, 2025 ▸). The structure of the Fe_2_ site is quite similar to the SCX structure (Fig. 2 ▸ and Table 2 ▸): Asp-84 shows monodentate binding, with one Fe—OE distance of 1.99–2.00 Å and the other of 2.97–3.04 Å (in the two chains). The strain energies are 124–478 kJ mol^−1^ with different *X* ligands. The two Fe—O_*X*_ distances are uneven, 1.88–1.93 and 1.77–1.84 Å, and slightly shorter than in the SCX structure. The Fe—Fe distance is 3.25 Å, also shorter than in the SCX structure.

The QR structures show the same trends as for the SCX structures (Fig. 2 ▸ and Table 2 ▸). The Fe—O_*X*_ distances increase when going from *X* = O^2−^ via OH^−^ to H_2_O, from 1.83–1.84 to 1.90–1.92 and 1.99–2.00 Å, respectively. There are only small differences between the QR structures and the original XFEL structure, with mean absolute differences of 0.03–0.05 Å for the first-sphere Fe–ligand distances (lowest for *X* = O^2−^ and highest for H_2_O). The Fe—Fe distance also increases somewhat with the *X* ligand, 3.24, 3.28 and 3.30 Å for O^2−^, OH^−^ and H_2_O, respectively.

**Figure 3:**
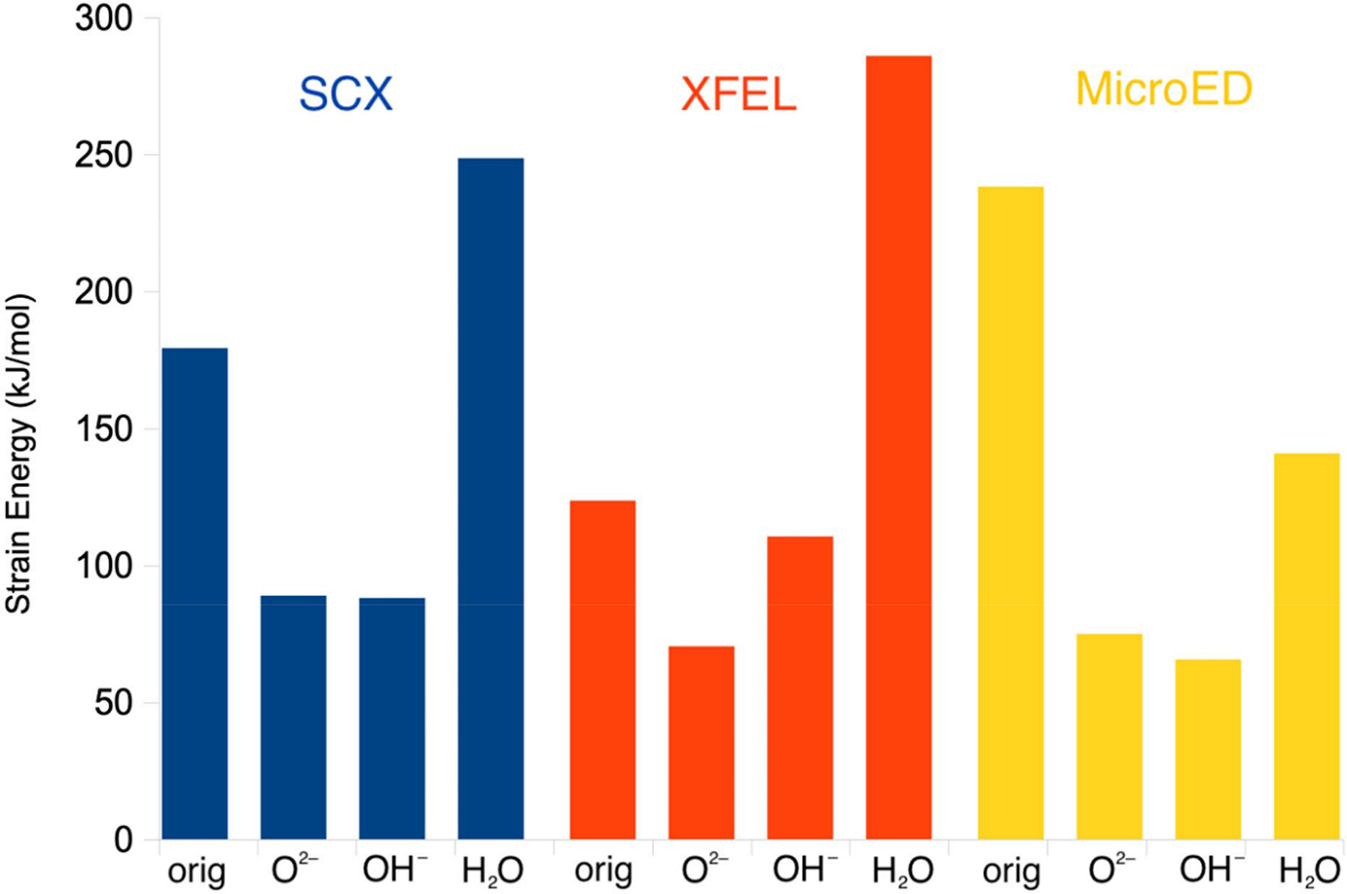
Strain energies of the various original and QR metR2a structures.

The structure with *X* = O^2−^ gives the lowest strain energy, 70 kJ mol^−1^, whereas the strain energy of the OH^−^ structure is somewhat larger (111 kJ mol^−1^) and that of the H_2_O structure is much larger (286 kJ mol^−1^) (Fig. 3 ▸). Likewise, the average RSZD score of the 11 residues in the QM system is slightly lower for *X* = O^2−^ (1.3) than for the other two structures (1.9 and 2.4 for *X* = OH^−^ and H_2_O, respectively). It is in particular the *X* ligand, the two Fe ions and the two water molecules that have improved the RSZD scores for the O^2−^ structure. The average RSZD score of the O^2−^ structure is the same as in the original (non-QR) structure. Thus, the QR results clearly show that the bridging ligand is O^2−^, in agreement with spectroscopic data (Sjöberg *et al.*, 1982 ▸; Bunker *et al.*, 1987 ▸; Scarrow *et al.*, 1986 ▸).

### 3.3. Oxidized MicroED structure

**Figure 4:**
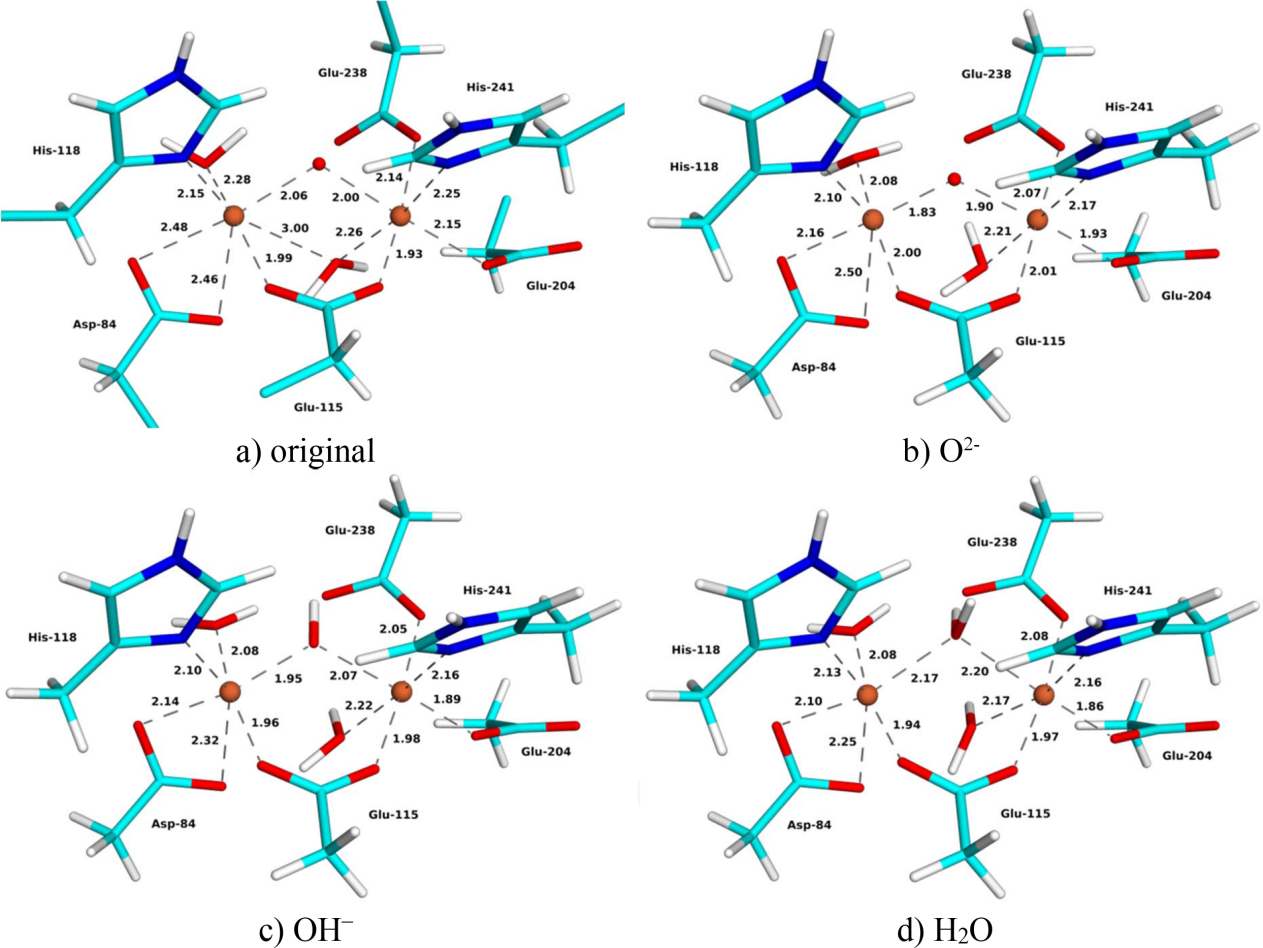
(*a*) Original and (*b*)–(*d*) QR MicroED structures with (*b*) *X* = O^2−^, (*c*) *X* = OH^−^ and (*d*) *X* = H_2_O of oxidized metR2a. Key distances are given in ångströms.

The MicroED structure of metR2a was obtained for this study and it is at 2.00 Å resolution. The Fe_2_ site is quite similar to that in the SCX structure [Fig. 4 ▸(*a*)] but there is still a 0.11 Å mean absolute difference in the 11 coordinating Fe—O/N distances (0.08 Å for the other subunit, Table 2 ▸). The two Fe—O distances of Asp-84 are more similar to each other in the MicroED structure, 2.21–2.48 and 2.46–2.62 Å, but the Fe—O_*X*_ distances are longer, 1.97–2.06 Å. The Fe—Fe distance is the same as in the SCX structure, 3.35–3.38 Å.

The three QR structures show the same trends as for the other two structures (Fig. 4 ▸ and Table 2 ▸). The Fe—O_*X*_ distances increase when going from *X* = O^2−^ via OH^−^ to H_2_O, 1.83–1.90 to 1.95–2.07 and 2.17–2.20 Å. Meanwhile, the distances to the other ligands mostly decrease, except for His-118, Glu-238 and the water molecules. The *X* = H_2_O structure could be obtained only with restraints on the O—H distance of the water ligand; otherwise a proton moved to Glu-238, forming a bridging OH^−^ ion. The Fe—Fe distance shows a larger variation than in the other QR structures, 3.34, 3.49 and 3.59 Å for O^2−^, OH^−^ and H_2_O, respectively.

The *X* = OH^−^ QR structure gives the lowest strain energy, 66 kJ mol^−1^, compared with 75 and 141 kJ mol^−1^ for the O^2−^ and H_2_O structures, respectively (Fig. 3 ▸). The same ranking was also observed for the average RSZD scores, 1.2 for OH^−^, compared with 1.5 and 1.4 for O^2−^ and H_2_O, respectively. The OH^−^ structure is best for Glu-115, Fe1, Fe2 and Wat2, whereas the H_2_O structure gives the lowest RSZD scores for Asp-84, Glu-204 and Wat1, and the O^2−^ structure is best for the other four ligands. The ligand *X* has the highest RSZD score in all structures, 2.2–2.6, but the score is lowest when *X* = O^2−^. The QR OH^−^ structure has a mean absolute deviation of 0.09 Å from the original ED structure (average over the two subunits) for the 11 short Fe—O/N distances in Table 2 ▸ (0.10 and 0.11 Å for the QR O^2−^ and H_2_O structures, respectively). In particular, the average Fe—O_*X*_ distance of the QR OH^−^ structure (2.01 Å) is much closer to that of the original structure (2.02 Å) than that of the QR structure with O^2−^ (1.86 Å). Altogether, QR points to OH^−^ as the most likely interpretation of the MicroED structure. This is in contrast to the experimental observation that oxidized metR2a should contain O^2−^ (Sjöberg *et al.*, 1982 ▸; Bunker *et al.*, 1987 ▸; Scarrow *et al.*, 1986 ▸), which indicates that the structure may have been photoreduced during data collection.

### 3.4. Reduced SCX structure

**Figure 5:**
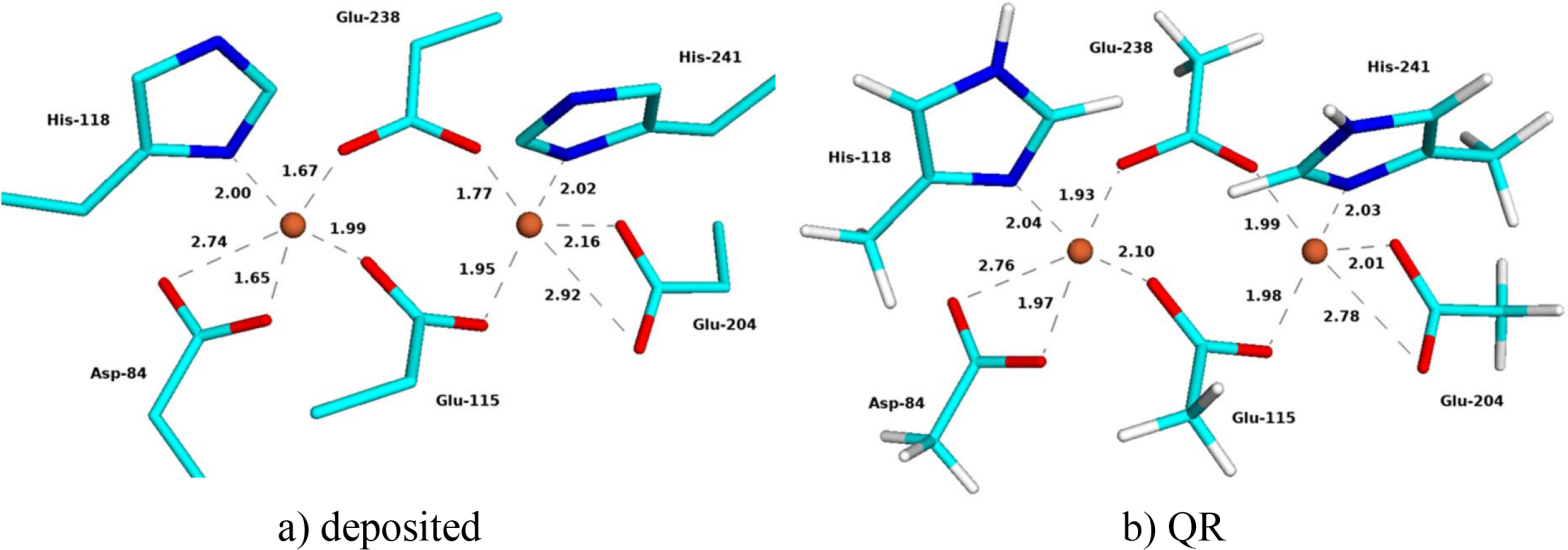
(*a*) Deposited and (*b*) QR SCX structures of reduced R2a. Key distances are given in ångströms.

The 1xik SCX structure is from 1996 and it is at 1.70 Å resolution (Logan *et al.*, 1996 ▸). The two Fe ions are both in the reduced 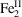 state. The reduced structure differs significantly from the oxidized state. Glu-204 now shows more bidentate binding to Fe2 [the two Fe—O distances are 2.16–2.40 and 2.51–2.92 Å; Fig. 5 ▸(*a*) and Table 3 ▸], whereas Glu-238 has moved to a bridging position. Moreover, none of the three solvent molecules in the oxidized structure are present in this structure. Asp-84 is still halfway between mono- and bidentate binding, with Fe—OE2 and Fe—OE1 distances of 1.65–1.96 and 2.73–2.74 Å in the two subunits, respectively (Table 3 ▸). Consequently, both metals are approximately five-coordinate. The Fe—O distances to the two bridging Glu ligands are 1.67–2.16 Å and the Fe—N distances to the two His ligands are 2.00–2.07 Å. There are quite extensive differences in the metal–ligand distances in the two subunits of the dimeric protein, and several of the reported distances are conspicuously short (down to 1.65 Å). The Fe—Fe distance is 3.88–3.94 Å, *i.e.* ∼0.5 Å larger than in the oxidized structure.

In the QR structure, the short Fe—O distances have been corrected [the shortest distance is 1.97 Å; Fig. 5 ▸(*b*) and Table 3 ▸]. Asp-84 and Glu-204 still bind halfway between mono- and bidentate with Fe—O bond lengths of 1.97–2.01 and 2.76–2.78 Å. The Fe—O distances for the two bridging Glu ligands are 1.93–2.10 Å, whereas the Fe—N distances to the two His ligands are 2.03–2.04 Å. The average RSZD score (4.4) is appreciably larger than that for the deposited structure (2.5), reflecting especially an increase in the score for Fe1 (which had two unrealistically short Fe—O distances; the RSZD score increases from 6.2 to 13.3). On the other hand, the strain energy for the QR structure (86 kJ mol^−1^) is appreciably lower than that for the deposited structure (365 kJ mol^−1^), showing that it is appreciably more chemically reasonable. This is the expected behaviour of QR. In the deposited structure, the metal site is essentially unrestrained, leading to some unrealistically short Fe—O bonds (in contrast to all the amino acids, for which empirical restraints apply, ensuring that all their bond lengths and angles make chemical sense). With QR, restraints are also added to the metal, directly correcting the erroneous bond lengths, but at the expense of increasing the RSZD scores (empirical restraints on the amino acids also increase the RSZD score and the *R* values). The QR structure differs significantly from the optimum vacuum structure, in which both Asp-84 and Glu-204 show bidentate binding. The high RSZD score of Fe1 in both the deposited and QR structures indicates that there is some problem with the structure that QR cannot fix, *e.g.* a mixture of several states.

We will see below that both the XFEL and MicroED structures show an additional solvent ligand, essentially in the position of Wat2 in the oxidized structures. However, there is no support for such a ligand from the electron density of the SCX structure. Still, we tried to add a water molecule near the position observed in the reduced XFEL and MicroED structures and ran QR with *w*_x_ = 1. This gave a structure that was virtually identical to the QR structure without the water molecule. The Fe–ligand distances change by less than 0.04 Å. The added water molecule binds weakly to Fe2, at a distance of 2.49 Å, whereas the distance to Fe1 is 3.11 Å. This gave a strongly increased strain energy of 154 kJ mol^−1^. Moreover, the RSZD score increased for all residues in the QM region except Fe1. In particular, the added water molecule has an RSZD score of 12.5, showing that it is not supported by the SCX raw data.

The reason for the difference in water binding is probably differences in the crystallization conditions (Pacoste, 2025 ▸). The earlier SCX structure was obtained in the presence of 1 m*M* ethylmercury thiosalicylate. Mercury binds to multiple sites in the protein, including the backbone C=O group of Cys-214. This interaction alters the orientation of the α-helix containing Cys-214, resulting in a repositioning of Phe-208 closer to the Fe_2_ centre, which restricts the space and probably prevents the accommodation of a water molecule. The XFEL and MicroED structures were obtained without mercury and can therefore be expected to be more representative of the physiological state.

### 3.5. Reduced XFEL structure

The XFEL structure of reduced redR2a (9sih) is at 1.7 Å resolution. The Fe_2_ site is rather similar to that in the SCX structure. Both Glu-115 and Glu-238 bridge the two Fe ions with Fe—O distances of 1.94–2.08 Å (Table 3 ▸). In chain *A*, Glu-238 is modelled with two conformations, one where it is bridging with one OE atom bound to each Fe ion, and one in which only OE2 bridges the two Fe ions with Fe—O distances of 2.26 and 2.08 Å, whereas OE1 binds only to Fe2 with an Fe—O distance of 2.01 Å (not shown in Table 3 ▸). Glu-204 binds bidentately to Fe2 with Fe—O distances of 2.16–2.31 Å, whereas Asp-84 shows a binding that is more monodentate with Fe—O distances of 1.97–1.99 and 2.76–2.79 Å. The two Fe—N distances are 2.02 and 2.22 Å. The Fe—Fe distance is only 3.21 Å, *i.e.* 0.7 Å shorter than in the SCX structure and actually 0.04 Å shorter than in the oxidized XFEL structure. As mentioned above, there is a water ligand binding to Fe2 with an Fe—O distance of 2.26–2.29 Å. The Fe1—O distance is 3.69 Å.

**Figure 6:**
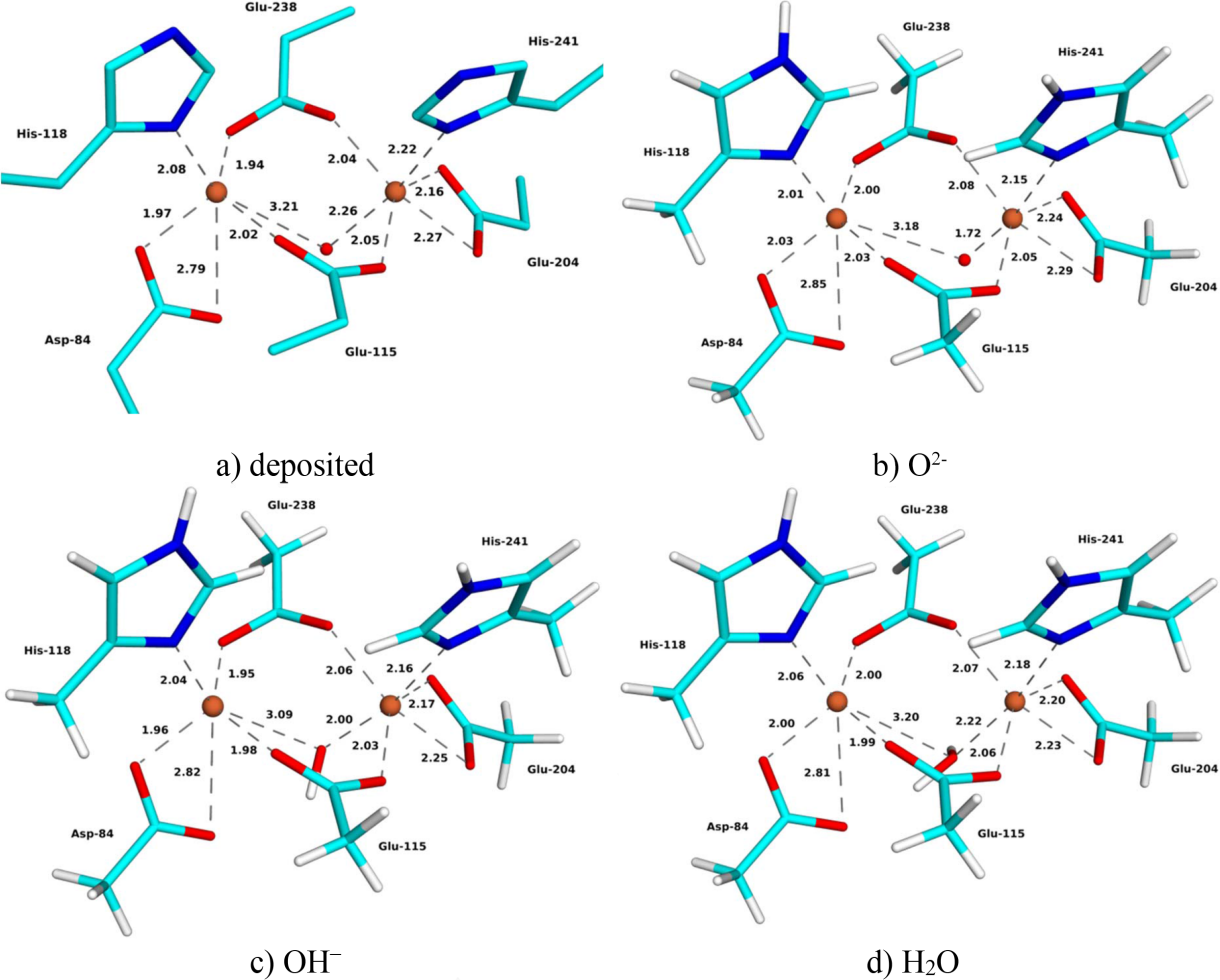
(*a*) Deposited and (*b*)–(*d*) QR XFEL structures with (*b*) *X* = O^2−^, (*c*) *X* = OH^−^ and (*d*) *X* = H_2_O of reduced R2a. Key distances are given in ångströms.

As for the oxidized structures, we performed QR for three possible interpretations of the solvent molecule *X*, O^2−^, OH^−^ and H_2_O. The QR structures behave the same way as for the oxidized structures (Fig. 6 ▸ and Table 3 ▸): the Fe2—O_*X*_ distances increase when going from *X* = O^2−^ via OH^−^ to H_2_O, 1.72, 2.00 and 2.22 Å, respectively. It remains far from Fe1 (3.09–3.20 Å). The Fe—Fe distance is constant and much longer than in the original structure, 3.67–3.69 Å. In all the QR structures, Glu-115 and Glu-238 bridge the two Fe ions without any tendency towards bidentate binding. Likewise, Glu-204 remains bidentate (2.17–2.29 Å) and Asp-84 mainly monodentate in all the QR structures (1.96–2.03 and 2.81–2.85 Å). The Fe—N bond lengths are 2.01–2.06 and 2.15–2.25 Å.

The structure with *X* = H_2_O gives the lowest strain energy (76 kJ mol^−1^) whereas those of the other two structures are much larger (193 kJ mol^−1^ for OH^−^ and 447 kJ mol^−1^ for O^2−^). Likewise, the average RSZD score of the nine residues in the QM system is considerably lower for *X* = H_2_O (1.5) than for the other two structures, 2.5 for OH^−^ and 3.4 for O^2−^. It is particularly the RSZD score of the *X* ligand that is improved. However, the RSZD scores of Glu-238 and Fe1 are also quite large for the H_2_O structure, indicating that Glu-238 may be disordered in chain *B*, explaining the unexpectedly short Fe—Fe distance in the original structure (the average RSZD score is slightly better than that for the best QR structure, 1.2). Still, the QR results clearly indicate that the reduced XFEL structure contains a water ligand.

### 3.6. Reduced MicroED structure

**Figure 7:**
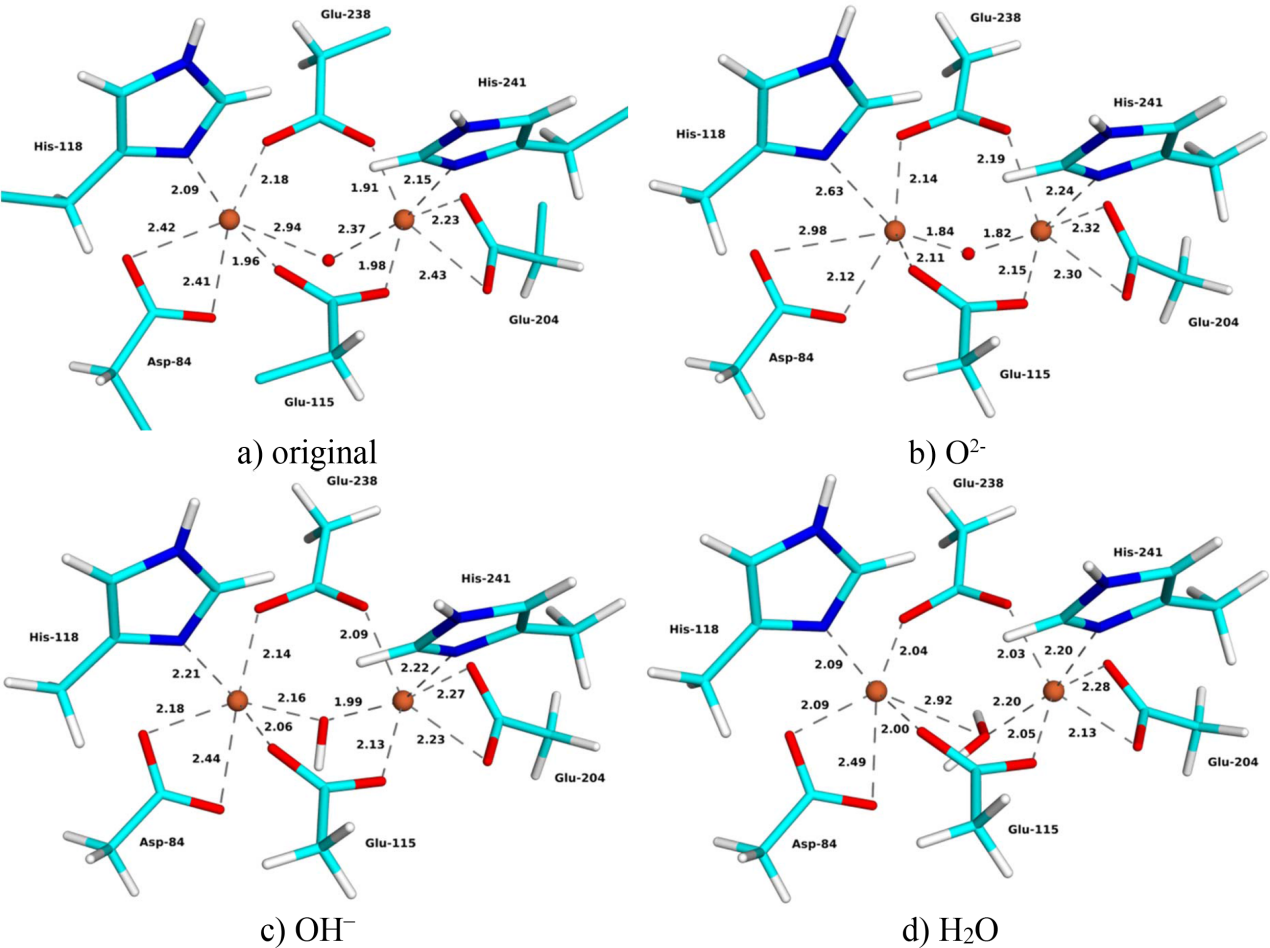
(*a*) Original and (*b*)–(*d*) QR MicroED structures with (*b*) *X* = O^2−^, (*c*) *X* = OH^−^ and (*d*) *X* = H_2_O of reduced R2a. Key distances are given in ångströms.

Finally, we considered the MicroED structure of the reduced dinuclear site. The structure was obtained for this study and it is at 2.20 Å resolution. In agreement with the other two reduced structures, Glu-115 and Glu-238 bridge the two Fe ions with Fe—O distances of 1.91–2.18 Å in the *B* subunit, but with longer and more varying distances in the *C* subunit, 2.15–2.78 Å [Fig. 7 ▸(*a*) and Table 3 ▸]. Asp-84 and Glu-204 both bind bidentately with rather similar Fe—O distances of 2.07–2.47 Å. There is a solvent molecule that is close to Fe2 (2.37–2.39 Å), but it is also rather close to Fe1 (2.94–2.99 Å). The Fe—Fe distance is 3.61–3.69 Å, *i.e.* intermediate between the SCX and XFEL structures.

Again, we performed QR for three possible interpretations of the solvent molecule (*X*), O^2−^, OH^−^ and H_2_O (in subunit *C*). The results are presented in Figs. 7 ▸(*b*)–7 ▸(*d*) and in Table 3 ▸. With *X* = O^2−^ and OH^−^, the solvent molecule bridges the two Fe ions with short Fe—O distances of 1.82–1.84 Å for O^2−^ and 1.99–2.16 Å for OH^−^. However, for *X* = H_2_O it takes a position similar to the original structure, with Fe—O distances of 2.20 and 2.92 Å. In all structures Glu-115 and Glu-238 bridge the two Fe ions with similar Fe—O distances of 2.00–2.19 Å. Glu-204 remains bidentate with Fe—O distances of 2.13–2.32 Å, whereas Asp-84 has one short and one long Fe—O bond of 2.09–2.18 and 2.44–2.98 Å, respectively. The two Fe—N distances are 2.09–2.21 Å for His-118 and 2.20–2.24 Å His-241, reflecting the higher coordination number of Fe2. However, with *X* = O^2−^, the Fe1—N bond becomes quite long, 2.63 Å, owing to the short Fe—O^2−^ bond in the *trans* position (1.84 Å).

The strain energies of the *X* = OH^−^ and H_2_O structures are quite low, 84 and 90 kJ mol^−1^, respectively, whereas the strain energy is much larger for the O^2−^ structure, 264 kJ mol^−1^. Likewise, the deviation from the optimum vacuum geometry is lower for the OH^−^ and H_2_O structures (0.07 Å) than for the O^2−^ structure (0.20 Å). On the other hand, the average RSZD score of the nine residues in the QM system is considerably lower for *X* = H_2_O (1.3) than for the other two structures (2.0 and 3.5). This is mainly caused by the RSZD scores of Fe1 and the *X* ligand. In fact, the average RSZD score of the H_2_O structure (2.2) is appreciably better than that for the original structure. Again, the QR results conclusively indicate that the reduced MicroED structure contains a water ligand.

## 4. Conclusions

In this study, we have presented the first quantum refinements of a protein employing XFEL and MicroED data. As the test case, we used six structures of oxidized and reduced ribonucleotide reductase R2a protein and concentrated on the binuclear Fe_2_ site. With our new implementation of QR in the *Phenix* software (Lundgren *et al.*, 2024 ▸), both XFEL and MicroED data could be used without any change in the code, using neutral-atom scattering factors (Colliex *et al.*, 2006 ▸) (QR can be applied to any method that is allowed and treated by *phenix.refine* or *phenix.real_space_refine*).

QR of the binuclear Fe_2_ site in R2a shows that the results obtained using data from the different radiation sources are similar and the trends are also similar. Variation of the *w*_x_ weight factor in equation (1) shows that QR behaves as expected for all three data sets (with small values of *w*_x_ the structure is biased towards QM, whereas with large *w*_x_ values it is biased towards the experimental data; Figs. S1 and S2). Ideal values of *w*_x_ determined as a compromise between the strain energies and the average RSZD scores (Lundgren *et al.*, 2024 ▸) coincided with the values determined by *Phenix* for standard refinement for all three methods. Therefore, in future applications of QR, no individual investigation of the *w*_x_ factor seems to be needed and the value suggested by *Phenix* can be used, *i.e.* the same procedure as was used with our previous im­plementation of QR with the *CNS* software (Ryde *et al.*, 2002 ▸).

For the oxidized metR2a state, the QR structures obtained with the three methods are quite similar. The largest difference is for the binding of Asp-84, which is bidentate in the MicroED structures but mainly monodentate in the SCX and XFEL structures, leading to variations of up to 0.36 and 0.56 Å in the Fe1—O_D84_ distances. Previous QM calculations have shown that the monodentate and bidentate binding modes of carboxylate groups to metals have similar energies and the two structures can easily interchange (Ryde, 1999 ▸). If this residue is excluded, the mean absolute deviation of the ten short Fe—O/N distances in Table 2 ▸ is 0.03–0.04 Å for the three QR structures with O^2−^ (least for SCX versus MicroED and most for MicroED versus XFEL). In fact, all three structures indicate that the Fe1—O_*X*_ distance is 0.01–0.09 Å shorter than the Fe2—O_*X*_ distance. Thus, all three methods seem to give comparable structures.

For the reduced structures, the largest difference in the QR structures is in the binding of Glu-204 to Fe2. In the SCX structure it binds monodentately, whereas in the other two structures the binding is bidentate. The SCX structure also differs considerably in terms of the missing solvent molecule. If Glu-204 is excluded, the mean absolute difference in the Fe—O/N bond lengths between the XFEL and MicroED structures is only 0.03 Å, whereas the SCX structure differs by 0.08–0.10 Å.

Notably, QR improves the structures in several aspects. In particular, it corrects unrealistically short Fe—O bonds (1.65–1.77 Å) in the redR2a SCX structure. More subtle improvements are obtained for the other structures. QR provides restraints for the metal sites from accurate QM calculations, including the correct oxidation and spin state of the metals, as well as the influence of all first-sphere ligands. In the original (non-QR) structures, the metal site is essentially unrestrained (besides a repulsive van der Waals potential), meaning that the geometry of the metal site is determined only by the experimental data. In contrast, all the amino acid parts of the structure are treated with standard empirical restraints to ensure chemically reasonable bond lengths, angles *etc.* Thus, QR is a powerful method to provide restraints for metal sites as well, giving them a similar (or better) accuracy to the protein.

Restraints will always move the structure slightly away from the experimental data (but for accurate experimental data and good QM methods they should nearly coincide). Therefore, it is expected that the RSZD values increase slightly in the QR structures. However, for all structures except redRa2 with SCX, the increase in the average RSZD score is marginal (0.05–0.2), and for the MicroED redR2a structure the average RSZD score actually decreases substantially. This is possible because the density maps are model biased and indicates that QR indeed provides improved structures.

Finally, we examined whether QR with the three sets of data might determine the nature of the bridging solvent molecule, by performing QR using three interpretations, *X* = O^2−^, OH^−^ or H_2_O, and evaluating the structures with respect to the strain energies and the RSZD scores. For the reduced state, we could unambiguously show that the XFEL and MicroED structures contain water and not OH^−^ or O^2−^ (the SCX structure does not contain any solvent ligand). QR also moves the solvent molecule to a more reasonable position in the MicroED structure.

For the oxidized structure, we could also unambiguously show that the bridging ligand is not water with all three data sets. In fact, with the MicroED data sets, we could obtain the water structures only if the protons were restrained to remain on the water oxygen. For the XFEL structure, the results indicate that the ligand is O^2−^, in agreement with spectroscopic data (Sjöberg *et al.*, 1982 ▸; Bunker *et al.*, 1987 ▸; Scarrow *et al.*, 1986 ▸). On the other hand, for the MicroED structure, there is a slight preference for *X* = OH^−^, which is seen already in the original refinement, giving Fe—O_*X*_ distances (1.97–2.06 Å) similar to what is expected for OH^−^ and appreciably longer than what is expected for O^2−^. The QR results confirm that this is a correct interpretation of the experimental data, indicating that the structure is photoreduced. For the SCX structure, the results cannot discern between O^2−^ and OH^−^, indicating that this structure is also partly photoreduced and therefore contains several different states. This is also reflected by the fact that, even in the QR structures (which reflect a compromise between QM and experimental data), the Fe2—O_*X*_ distances for the O^2−^ structures are 0.06–0.08 Å shorter in the XFEL structure than in the other two structures.

In conclusion, we have shown that QR with XFEL and MicroED data is possible with the *QRef* interface and that reasonable results are obtained with the same discriminatory power as standard X-ray data. In the future, we will compare the current MicroED data with SerialED data (Pacoste, 2025 ▸; Hofer *et al.*, 2025 ▸) and study whether electron diffraction data from macromolecules may be used with more advanced (non-spherical) scattering factors that also consider partial charges for each species, and whether this approach can be used to observe protons more directly.

## Supplementary Material

Additional tables and figures. DOI: 10.1107/S1600576725011264/ei5140sup1.pdf

MicroED data set for the oxidised structure: https://doi.org/10.5281/zenodo.15783462

MicroED data set for the reduced structure: https://doi.org/10.5281/zenodo.15783683

## Figures and Tables

**Table 1:** Data merging and refinement statistics for MicroED data of metR2a and redR2a Statistics for the highest-resolution shell are shown in parentheses.

Sample	metR2a	redR2a
No. of merged datasets	22	69
Space group	*P*2_1_2_1_2_1_	*P*2_1_2_1_2_1_
Unit cell		
*a*	73.95	73.95
*b*	76.54	76.54
*c*	145.74	145.74
Resolution	20.18–2.00 (2.06–2.00)	19.76–2.20 (2.28–2.20)
No. of reflections		
Total	1689424 (30021)	4549469 (321805)
Unique	50161 (4248)	37677 (3719)
Multiplicity	33.66 (10.11)	120.74 (115.88)
*I*/σ(*I*)	5.73 (0.66)	10.22 (2.25)
*R*_meas_ (%)	48.1 (293.9)	53.8 (318.9)
CC_1/2_ (%)	98.4 (9.1)	97.8 (13.8)
Completeness (%)	88.10 (70.34)	85.81 (79.32)
No. of reflections used	49658 (3 909)	36701 (3 334)
*R*_work_/*R*_free_ (%)	22.22/25.28 (34.32/38.14)	21.64/26.39 (29.12/32.54)
RMSD bond lengths (Å)	0.004	0.003
RMSD bond angles (°)	0.53	0.52
Ramachandran		
Favoured (%)	96.60	97.93
Allowed (%)	2.51	1.92
Outliers (%)	0.89	0.15
Rotamer outliers	1.10	1.43
Clash score	6.41	5.21
Average *B* factor	42.27	38.51
Macromolecule	42.03	38.47
Ligands	24.52	29.70
Solvent	46.44	39.37

**Table 2:** Strain energies (kJ mol^−1^), average RSZD scores for the 11 residues in the QM system (individual data shown in Table S3) and Fe–ligand distances (Å) for the original structures (with two different polypeptide chains) and the QR structures with *X* = O^2−^, OH^−^ or H_2_O for metR2a Two distances are given for Asp-84 (D84), one for OD1 and the other for OD2.

			Fe1							Fe2					
*X*	Strain	RSZD	D84		E115	H118	W1	*X*	Fe2	E115	E204	E238	H241	W2	*X*
SCX															
Chain *A*	≥179†	2.5	2.86	1.98	2.02	2.12	2.30	1.93	3.38	2.04	1.96	2.06	2.22	2.30	1.94
Chain *B*			2.87	2.06	2.04	2.11	2.25	1.95	3.35	2.00	2.07	2.03	2.19	2.22	1.86
O^2−^	89	2.7	2.86	2.00	2.02	2.10	2.20	1.83	3.37	2.03	1.93	2.05	2.18	2.26	1.92
OH^−^	88	2.7	2.88	1.93	1.98	2.09	2.21	1.94	3.40	2.02	1.89	2.04	2.18	2.23	2.01
H_2_O	249	3.5	2.84	1.89	1.95	2.09	2.22	2.08	3.42	2.00	1.89	2.05	2.18	2.20	2.09

XFEL															
Chain *A*	≥124‡	1.3	3.04	2.00	2.01	2.08	2.20	1.93	3.25	2.08	1.97	2.02	2.15	2.29	1.77
Chain *B*			2.97	1.99	2.05	2.08	2.22	1.88	3.25	2.08	2.04	2.04	2.23	2.21	1.84
O^2−^	70	1.3	3.04	2.00	1.99	2.04	2.17	1.83	3.24	2.07	1.93	2.02	2.14	2.22	1.84
OH^−^	111	1.9	3.03	1.96	1.98	2.03	2.13	1.90	3.28	2.05	1.91	2.01	2.13	2.21	1.92
H_2_O	286	2.4	3.03	1.94	1.97	2.04	2.14	2.00	3.30	2.04	1.91	2.02	2.14	2.20	1.99

MicroED															
Chain *C*	≥238§	1.1	2.46	2.48	1.99	2.15	2.28	2.06	3.38	1.93	2.15	2.14	2.25	2.26	2.00
Chain *B*			2.62	2.21	2.00	2.16	2.30	1.97	3.35	2.00	2.05	2.01	2.28	2.09	2.02
O^2−^	75	1.5	2.50	2.16	2.00	2.10	2.08	1.83	3.34	2.01	1.93	2.07	2.17	2.21	1.90
OH^−^	66	1.2	2.32	2.14	1.96	2.10	2.08	1.95	3.49	1.98	1.89	2.05	2.16	2.22	2.07
H_2_O	141	1.4	2.25	2.10	1.94	2.13	2.08	2.17	3.59	1.97	1.86	2.08	2.16	2.17	2.20

† The strain energies are 183, 179 and 435 kJ mol^−1^ for O^2−^, OH^−^ and H_2_O, respectively.

‡ The strain energies are 124, 182 and 478 kJ mol^−1^ for O^2−^, OH^−^ and H_2_O, respectively.

§ The strain energies are 288, 238 and 393 kJ mol^−1^ for O^2−^, OH^−^ and H_2_O, respectively.

**Table 3:** Strain energies (kJ mol^−1^), average RSZD scores for the nine residues in the QM system (individual data shown in Table S4) and Fe–ligand distances (Å) for the original structures (with two different polypeptide chains for SCX and MicroED) and the best QR structures with *X* = O^2−^, OH^−^ or H_2_O for redR2a Two distances are given for Asp-84 (D84), one for OD1 and the other for OD2. The same applies for Glu-204 (E204; OE1 and OE2).

			Fe1							Fe2					
*X*	Strain	RSZD	D84		E115	H118	E238	*X*	Fe2	E115	E204		E238	H241	*X*
SCX															
Chain *A*	365	2.5	1.65	2.74	1.99	2.00	1.67		3.94	1.95	2.16	2.92	1.77	2.02	
Chain *B*			1.96	2.73	2.16	2.07	1.78		3.88	2.13	2.40	2.51	1.80	2.04	
None	86	4.4	1.97	2.76	2.10	2.04	1.93		3.92	1.98	2.01	2.78	1.99	2.03	
H_2_O	154	4.7	1.97	2.74	2.11	2.03	1.92	3.11	3.91	2.01	1.99	2.84	1.98	2.04	2.49

XFEL															
Chain *A*		1.2	2.76	1.99	2.08	2.02	2.03†	3.69	3.21	2.05	2.20	2.31	1.99†	2.22	2.29
Chain *B*	≥122‡		2.79	1.97	2.02	2.08	1.94	3.69	3.21	2.05	2.16	2.27	2.04	2.22	2.26
O^2−^	447	3.5	2.85	2.03	2.03	2.01	2.00	3.18	3.68	2.05	2.24	2.29	2.08	2.15	1.72
OH^−^	193	2.0	2.82	1.96	1.98	2.04	1.95	3.09	3.67	2.03	2.17	2.25	2.06	2.16	2.00
H_2_O	76	1.3	2.81	2.00	1.99	2.06	2.00	3.20	3.69	2.06	2.20	2.23	2.07	2.18	2.22

MicroED															
Chain *C*	≥214§	2.2	2.41	2.42	1.96	2.09	2.18	2.94	3.61	1.98	2.23	2.43	1.91	2.15	2.37
Chain *B*			2.47	2.46	2.15	2.05	2.52	2.99	3.69	2.26	2.39	2.07	2.78	2.31	2.39
O^2−^	264	3.5	2.98	2.12	2.11	2.63	2.14	1.84	2.99	2.15	2.32	2.30	2.19	2.24	1.82
OH^−^	84	2.0	2.44	2.18	2.06	2.21	2.14	2.16	3.37	2.13	2.27	2.23	2.09	2.22	1.99
H_2_O	90	1.3	2.49	2.09	2.00	2.09	2.04	2.92	3.57	2.05	2.28	2.13	2.03	2.20	2.20

† In the original structure, Glu-238 has two conformations, of which only conformation *A* is shown in the table. In conformation *B*, OE2 bridges the two Fe ions with Fe—O distances of 2.26 and 2.08 Å, whereas OE1 binds only to Fe2 with an Fe—O distance of 2.01 Å.

‡ The strain energies are 706, 301 and 112 kJ mol^−1^ for O^2−^, OH^−^ and H_2_O, respectively.

§ The strain energies are 771, 361 and 214 kJ mol^−1^ for O^2−^, OH^−^ and H_2_O, respectively.

## Data Availability

All MicroED datasets have been deposited on Zenodo and are available for metR2a (https://zenodo.org/records/15783462) and redR2a (https://zenodo.org/records/15783683).
