# Supplementary material for: Quantum refinement with electron diffraction and X-ray free-electron laser data: comparative study of ribonucleotide reductase dimetal site
Source: J Appl Crystallogr. 2026 Feb 9;59(Pt 2):277–90. doi: 10.1107/S1600576725011264 (PMC13060455; doi:10.1107/S1600576725011264)
Supplement: Supplementary file 1 [file j-59-00277-sup1.pdf]

JOURNAL OF APPLIED CRYSTALLOGRAPHY

Volume 59 (2026)

Supporting information for article:

Quantum refinement with electron diffraction and X-ray free-electron laser data: comparative study of ribonucleotide reductase dimetal site

Kristoffer J. M. Lundgren, Xiaoli Sun, Laura Pacoste, Rohit Kumar, Gerhard Hofer, Hongyi Xu, Xiaodong Zou, Martin Högbom, Esko Oksanen and Ulf Ryde

Table S1. Beam settings and data collection parameters per dataset used in the final merge of the metR2a MicroED data. The datasets are numbered in the same way as on Zenodo.

Data set	Tilt step °/frame	Exposure time s	No. frames	Tilt range °	Flux $eÅ^{-2}s^{-1}$	Fluence $eÅ^{-2}$	C2 µm	CL m	Beam size µm	Spot size
1	0.5	0.5	129	64.5	0.21	13.3	50	1.5	3	8
2	0.5	1	199	99.5	0.01	2.0	20	1.2	2.5	8
3	0.5	0.5	199	99.5	0.01	1.0	20	1.2	2.5	8
4	0.5	0.5	199	99.5	0.01	1.0	20	1.2	2.5	8
5	0.5	1	199	99.5	0.01	2.0	20	1.2	2.5	8
6	0.2	0.5	99	19.8	0.21	10.2	50	1.5	3	8
7	0.2	0.5	99	19.8	0.21	10.2	50	1.5	3	8
8	0.2	0.5	99	19.8	0.05	2.6	50	1.5	3	10
9	0.2	0.5	99	19.8	0.05	2.6	50	1.5	3	10
10	0.2	0.5	99	19.8	0.05	2.6	50	1.5	3	10
11	0.2	0.5	99	19.8	0.05	2.6	50	1.5	3	10
12	0.2	0.5	99	19.8	0.05	2.6	50	1.5	3	10
13	0.2	0.5	99	19.8	0.05	2.6	50	1.5	3	10
14	0.2	0.5	99	19.8	0.05	2.6	50	1.5	3	10
15	0.2	0.5	99	19.8	0.21	10.2	50	1.5	3	8
16	0.2	0.5	99	19.8	0.21	10.2	50	1.5	3	8
17	0.2	0.5	99	19.8	0.21	10.2	50	1.5	3	8
18	0.2	0.5	99	19.8	0.21	10.2	50	1.5	3	8
19	0.2	0.5	99	19.8	0.21	10.2	50	1.5	3	8
20	0.2	0.5	99	19.8	0.05	2.6	50	1.5	3	10
21	0.5	0.5	159	79.5	0.21	16.4	50	1.5	3	8
22	0.5	0.5	159	79.5	0.21	16.4	50	1.5	3	8

Table S2. Beam settings and data collection parameters per dataset used in the final merge of the metR2a MicroED data. The datasets are numbered in the same way as on Zenodo.

Data set	Tilt step °/frame	Exposure time s	No. frames	Tilt range °	Flux eÅ^{-2} s^{-1}	Fluence eÅ^{-2}	C2 µm	CL m	Beam size µm	Spot size
1	1	1	39	39	0.19	7.5	20	1.35	1.5	8
2	1	1	59	59	0.19	11.3	20	1.35	1.5	8
3	1	1	50	50	0.19	9.6	20	1.35	1.5	8
4	1	1	45	45	0.19	8.6	20	1.35	1.5	8
5	1	1	40	40	0.19	7.7	20	1.35	1.5	8
6	1	1	39	39	0.19	7.5	20	1.35	1.5	8
7	1	1	30	30	0.19	5.8	20	1.35	1.5	8
8	1	1	30	30	0.19	5.8	20	1.35	1.5	8
9	1	1	35	35	0.19	6.7	20	1.35	1.5	8
10	1	1	38	38	0.19	7.3	20	1.35	1.5	8
11	1	1	39	39	0.19	7.5	20	1.35	1.5	8
12	1	1	30	30	0.19	5.8	20	1.35	1.5	8
13	1	1	45	45	0.19	8.6	20	1.35	1.5	8
14	1	1	45	45	0.19	8.6	20	1.35	1.5	8
15	1	1	45	45	0.19	8.6	20	1.35	1.5	8
16	1	1	45	45	0.19	8.6	20	1.35	1.5	8
17	1	1	45	45	0.19	8.6	20	1.35	1.5	8
18	1	1	39	39	0.19	7.5	20	1.35	1.5	8
19	1	1	39	39	0.19	7.5	20	1.35	1.5	8
20	1	1	45	45	0.19	8.6	20	1.35	1.5	8
21	1	1	45	45	0.19	8.6	20	1.35	1.5	8
22	1	1	40	40	0.19	7.7	20	1.35	1.5	8
23	1	1	45	45	0.19	8.6	20	1.35	1.5	8
24	1	1	45	45	0.19	8.6	20	1.35	1.5	8
25	1	1	39	39	0.19	7.5	20	1.35	1.5	8
26	1	1	49	49	0.19	9.4	20	1.35	1.5	8
27	1	1	45	45	0.19	8.6	20	1.35	1.5	8
28	1	1	45	45	0.19	8.6	20	1.35	1.5	8
29	1	1	20	20	0.19	3.8	20	1.35	1.5	8
30	1	1	28	28	0.19	5.4	20	1.35	1.5	8
31	1	1	39	39	0.19	7.5	20	1.35	1.5	8
32	1	1	39	39	0.19	7.5	20	1.35	1.5	8
33	1	1	45	45	0.19	8.6	20	1.35	1.5	8
34	1	1	45	45	0.19	8.6	20	1.35	1.5	8
35	1	1	30	30	0.19	5.8	20	1.35	1.5	8
36	1	1	30	30	0.19	5.8	20	1.35	1.5	8
37	1	1	30	30	0.19	5.8	20	1.35	1.5	8
38	1	1	35	35	0.19	6.7	20	1.35	1.5	8
39	1	1	30	30	0.19	5.8	20	1.35	1.5	8
40	1	1	35	35	0.19	6.7	20	1.35	1.5	8
41	1	1.5	39	39	0.19	11.2	20	1.35	1.5	8
42	1	1.5	30	30	0.19	8.6	20	1.35	1.5	8
43	1	1.5	40	40	0.19	11.5	20	1.35	1.5	8
44	1	1.5	39	39	0.19	11.2	20	1.35	1.5	8

Table S2 continued.

Data set	Tilt step °/frame	Exposure time s	No. frames	Tilt range °	Flux eÅ^{-2} s^{-1}	Fluence eÅ^{-2}	C2 µm	CL m	Beam size µm	Spot size
45	1	1.5	55	55	0.19	15.8	20	1.35	1.5	8
46	1	1.5	30	30	0.19	8.6	20	1.35	1.5	8
47	1	1.5	35	35	0.19	10.1	20	1.35	1.5	8
48	1	1.5	35	35	0.19	10.1	20	1.35	1.5	8
49	1	1.5	49	49	0.19	14.1	20	1.35	1.5	8
50	1	1	40	40	0.19	7.7	20	1.35	1.5	8
51	1	1.5	45	45	0.19	12.9	20	1.35	1.5	8
52	1	1.5	45	45	0.19	12.9	20	1.35	1.5	8
53	1	1	40	40	0.19	7.7	20	1.35	1.5	8
54	1	1	35	35	0.19	6.7	20	1.35	1.5	8
55	1	1	25	25	0.19	4.8	20	1.35	1.5	8
56	1	1	50	50	0.19	9.6	20	1.35	1.5	8
57	1	1	45	45	0.19	8.6	20	1.35	1.5	8
58	1	1	45	45	0.19	8.6	20	1.35	1.5	8
59	1	1	45	45	0.19	8.6	20	1.35	1.5	8
60	1	1	45	45	0.19	8.6	20	1.35	1.5	8
61	1	1	35	35	0.19	6.7	20	1.35	1.5	8
62	1	1	30	30	0.19	5.8	20	1.35	1.5	8
63	1	1	39	39	0.19	7.5	20	1.35	1.5	8
64	1	1	30	30	0.19	5.8	20	1.35	1.5	8
65	1	1.5	30	30	0.19	8.6	20	1.35	1.5	8
66	1	1.5	30	30	0.19	8.6	20	1.35	1.5	8
67	1	1.5	49	49	0.19	14.1	20	1.35	1.5	8
68	1	1.5	18	18	0.19	5.2	20	1.35	1.5	8
69	1	1.5	40	40	0.19	11.5	20	1.35	1.5	8

Table S3. Mean absolute deviation of the 12 Fe–ligand distances in Table 2 from the corresponding QM-optimised structure (Δr in Å), individual RSZD scores, R_{work} and R_{free} scores for the original structures and the best QR structures with X = O^{2-}, OH^- or H_2O for the metR2a structures.

	Δr	RSZD											R_{work}	R_{free}
	(Å)	D84	E115	H 118	E204	E238	H241	Fe1	Fe2	W1	W2	X		
SCX														
deposited	0.07	2.0	2.2	0.8	0.2	2.2	1.5	5.6	4.2	4.2	2.3	2.4	0.1607	0.1630
O^{2-}	0.04	2.3	2.0	1.6	0.7	2.1	1.8	7.1	4.0	3.2	2.4	2.7	0.1609	0.1636
OH^-	0.03	2.5	3.0	1.5	1.3	2.0	1.8	4.3	3.8	3.2	3.7	2.7	0.1608	0.1633
H_2O	0.06	2.6	3.1	1.7	1.9	1.8	1.8	5.9	5.1	3.9	5.8	5.3	0.1609	0.1632
XFEL														
original	0.08	0.2	0.7	1.0	2.4	1.5	2.3	0.9	0.2	2.1	2.1	0.9	0.1508	0.1853
O^{2-}	0.06	0.7	0.9	1.9	2.7	1.9	0.5	1.9	0.2	1.1	1.3	1.7	0.1535	0.1861
OH^-	0.05	0.6	0.9	2.0	2.9	1.8	0.5	2.2	1.8	1.9	1.9	3.9	0.1535	0.1860
H_2O	0.09	0.5	0.6	1.9	2.8	1.6	0.5	2.4	3.2	1.7	2.5	8.3	0.1537	0.1861
MicroED														
original	0.15	1.6	0.8	0.3	0.4	0.7	0.4	0.7	1.5	2.9	2.7	0.5	0.1899	0.2270
O^{2-}	0.05	2.2	1.4	0.5	0.5	0.5	1.2	2.5	1.3	2.2	2.6	1.4	0.1849	0.2304
OH^-	0.05	1.7	1.3	0.6	0.3	0.7	1.4	1.2	1.0	2.6	1.6	0.9	0.1851	0.2303
H_2O	0.05	1.6	1.5	1.1	0.2	0.7	1.3	1.8	1.8	2.6	1.3	1.8	0.1852	0.2312

Table S4. Mean absolute deviation of the 10 Fe–ligand distances in Table 3 from the corresponding QM-optimised structure (Δr in Å), individual RSZD scores, R_{work} and R_{free} scores for the original structures and the best QR structures with X = O^{2-}, OH^{-} or H_2O for redR2a.

	Δr	RSZD									R_{work}	R_{free}
	(Å)	D84	E115	H 118	E204	E238	H241	Fe1	Fe2	X		
SCX												
deposited	0.29	1.8	1.2	2.7	0.9	2.2	2.3	6.2	2.5		0.1880	0.1867
no	0.19	2.7	2.3	3.5	2.8	3.9	2.9	13.3	4.0		0.1873	0.1919
H_2O	0.18	3.3	4.1	3.5	3.1	4.0	3.2	12.3	4.0	12.5	0.1875	0.1925
XFEL												
chain B	0.13	0.6	0.8	0.6	0.3	1.7	2.0	2.1	1.4	1.2	0.1579	0.1905
O^{2-}	0.10	1.2	0.8	1.0	1.6	2.4	1.1	1.3	5.8	15.3	0.1612	0.1929
OH^{-}	0.15	1.0	1.3	0.9	0.8	2.5	1.0	3.6	5.3	6.4	0.1609	0.1919
H_2O	0.11	0.8	0.8	1.4	0.5	2.5	1.2	3.6	1.8	0.9	0.1608	0.1917
MicroED												
original	0.21	2.6	2.3	2.0	1.2	0.9	2.5	2.0	1.5	5.2	0.1853	0.2373
O^{2-}	0.20	1.2	1.0	2.4	1.1	1.1	1.7	14.4	0.9	7.7	0.1795	0.2410
OH^{-}	0.07	1.3	0.3	2.3	1.4	1.0	1.5	2.4	1.3	6.2	0.1793	0.2399
H_2O	0.07	1.9	0.2	2.2	1.1	1.5	1.4	0.6	1.2	1.9	0.1790	0.2410

Table S5. Strain energies (Str in kJ/mol), average RSZD scores for the 11 residues in the QM system and Fe–ligand distances (Å) for the QR XFEL structures of metR2a with X = O^{2-}, obtained at three QM levels of theory: 1: TPSS-D4/def2-SV(P) in vacuum, 2: the same method in a CPCM continuum solvent with a dielectric constant of 4, and 3: B3LYP-D4/def2-TZVP in vacuum. Two distances are given for Asp-84 (D84), one for OD1 and the other for OD2.

QM	Str	RSZD	Fe1							Fe2					
			D84		E115	H118	W1	X	Fe2	E115	E204	E238	H241	W2	X
1	70	1.3	3.04	2.00	1.99	2.04	2.17	1.83	3.24	2.07	1.93	2.02	2.14	2.22	1.84
2	33	1.3	3.06	2.00	2.00	2.02	2.17	1.82	3.25	2.07	1.97	2.02	2.13	2.24	1.83
3	54	1.3	3.05	1.99	2.01	2.04	2.19	1.80	3.25	2.07	1.94	2.02	2.15	2.24	1.87

Figure S1. Dependence of the strain energy (ΔE) and RSZD on the w_x weight factor for SCX (top), XFEL (middle) and MicroED (bottom) for X = O^{2-}, OH^- or H_2O for metR2a. The value of w_x was selected when the strain energy starts to increase but not to more than approximately twice the value at low w_x and the average RSZD have decreased below 3, viz. w_x = 1 for SCX and MicroED, and 3 for XFEL.

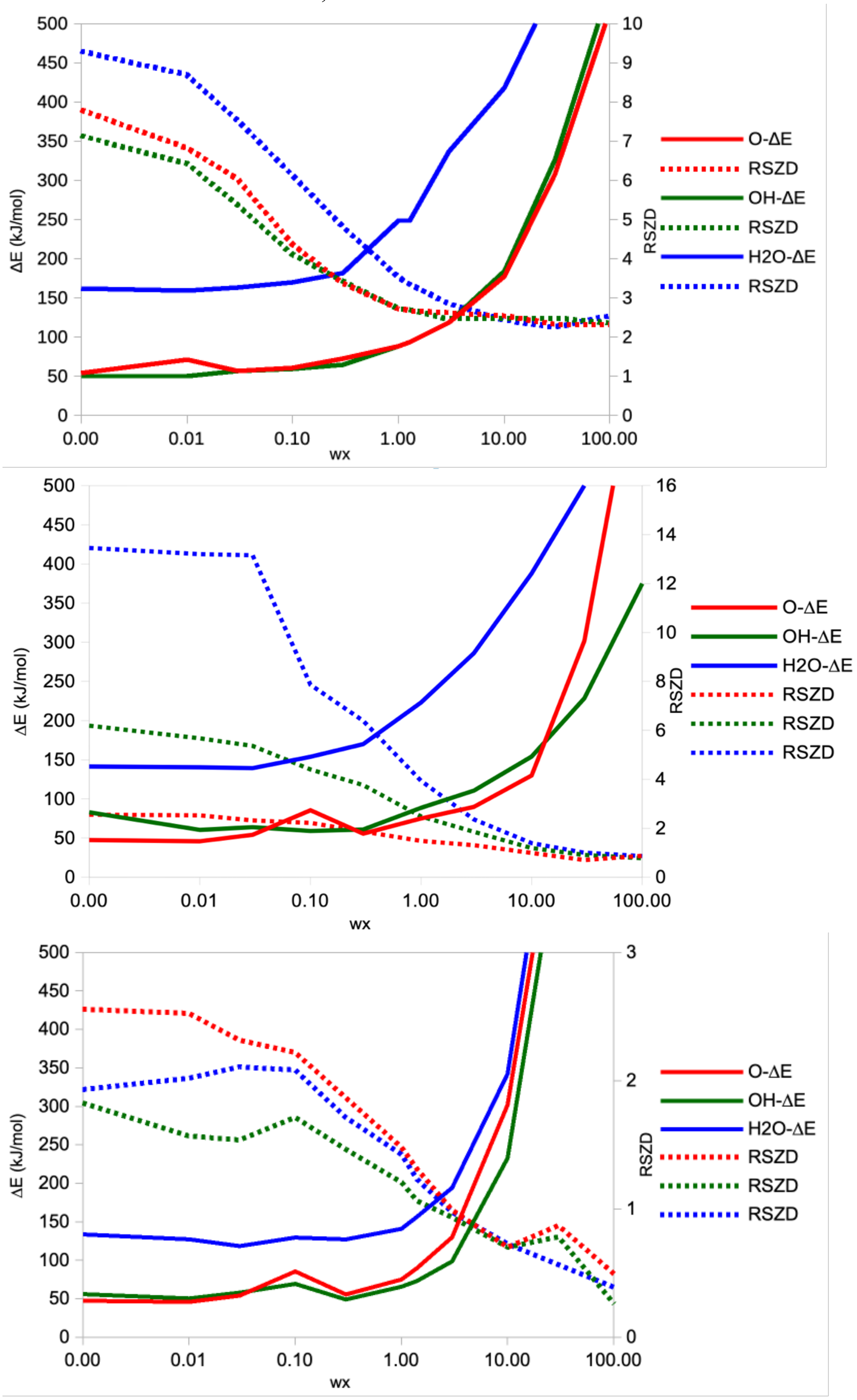

Figure S2. Dependence of the strain energy (ΔE) and RSZD on the w_x weight factor for SCX (top), XFEL (middle) and MicroED (bottom) for X = O^{2-}, OH^- or H_2O for redR2a. w_x was selected when the strain energy starts to increase but not to more than approximately twice the value at low w_x and the average RSZD have decreased below 3, viz. w_x = 1 for SCX and MicroED, and 3 for XFEL.

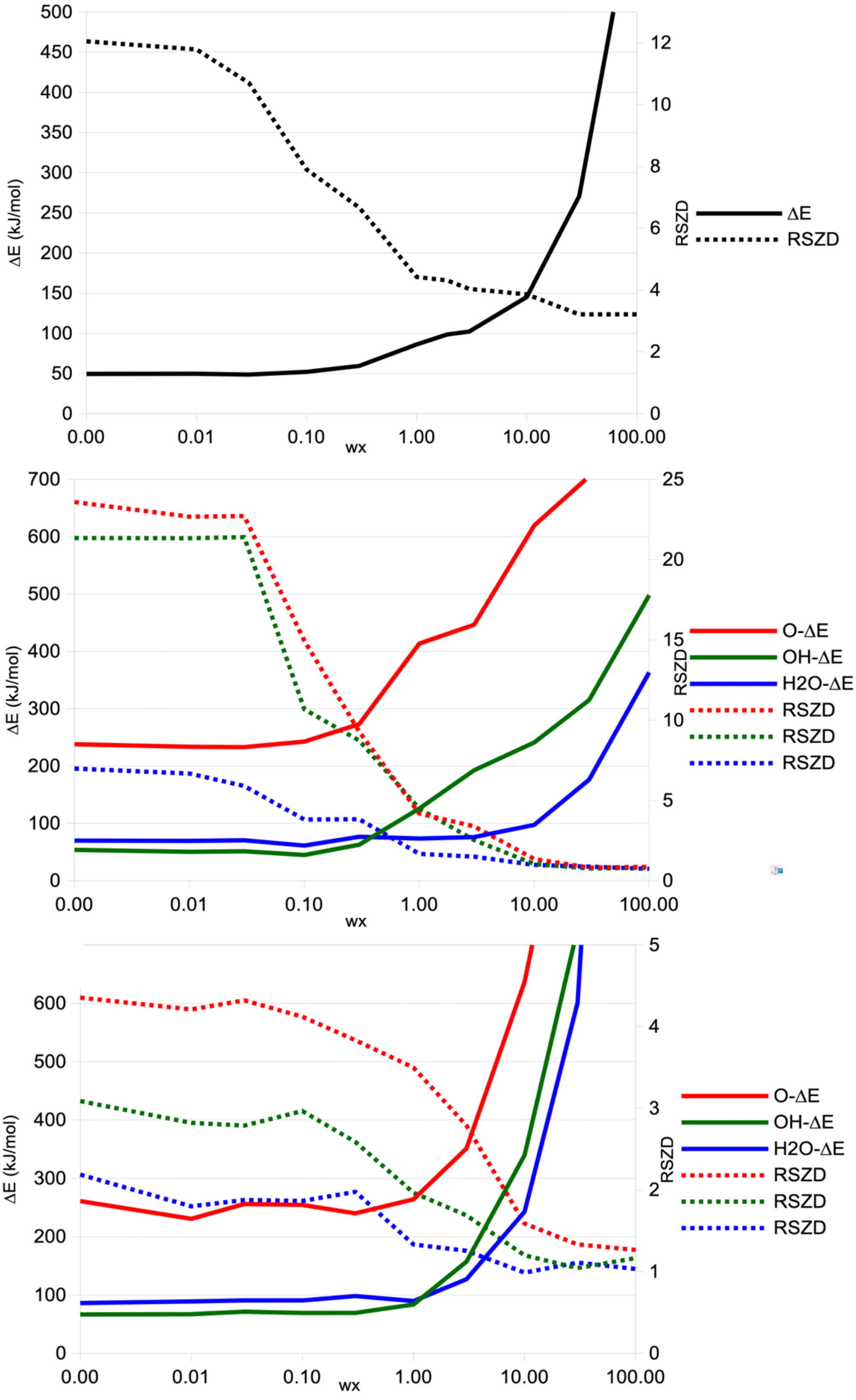

Table S6. Coordinates of the QM region for the best QR structures for each of the six structures.

```
REMARK Oxidised SCX structure, O2- ligand, wx=1
REMARK /home/ulf/Cosmos/Kristoffer/RNR/1mxr/o/wx/1/geo_opt_qm_constrained
ATOM    718  CA  ASP A  84       4.924   5.728  50.710  1.00 11.10           H
ATOM    721  CB  ASP A  84       5.293   6.739  50.948  1.00 12.21           C
ATOM    722  CG  ASP A  84       4.738   7.709  49.898  1.00 15.79           C
ATOM    723  OD1 ASP A  84       4.054   7.318  48.922  1.00 15.35           O
ATOM    724  OD2 ASP A  84       5.058   8.951  50.084  1.00 16.25           O
ATOM    725  HB2 ASP A  84       4.984   7.047  51.964  1.00 13.52           H
ATOM    726  HB3 ASP A  84       6.399   6.749  50.913  1.00 12.46           H
ATOM    968  CB  GLU A 115       8.110   6.908  46.769  1.00 10.52           H
ATOM    969  CG  GLU A 115       7.480   7.154  45.896  1.00 10.72           C
ATOM    970  CD  GLU A 115       6.300   8.031  46.262  1.00 12.41           C
ATOM    971  OE1 GLU A 115       6.294   8.559  47.424  1.00 13.52           O
ATOM    972  OE2 GLU A 115       5.402   8.161  45.379  1.00 10.24           O
ATOM    973  HG2 GLU A 115       8.089   7.686  45.141  1.00 10.18           H
ATOM    974  HG3 GLU A 115       7.112   6.220  45.425  1.00 10.71           H
ATOM    991  CA  HIS A 118       8.594  10.483  51.571  1.00  9.94           H
ATOM    994  CB  HIS A 118       7.711  10.291  50.939  1.00 10.29           C
ATOM    995  CG  HIS A 118       7.629  11.271  49.816  1.00 10.89           C
ATOM    996  ND1 HIS A 118       6.628  11.259  48.847  1.00 10.99           N
ATOM    997  CD2 HIS A 118       8.474  12.314  49.516  1.00 10.95           C
ATOM    998  CE1 HIS A 118       6.859  12.253  48.002  1.00 11.62           C
ATOM    999  NE2 HIS A 118       7.975  12.923  48.378  1.00 11.45           N
ATOM   1000  HB2 HIS A 118       7.775   9.262  50.543  1.00 12.15           H
ATOM   1001  HB3 HIS A 118       6.798  10.321  51.562  1.00 12.71           H
ATOM   1002  HD2 HIS A 118       9.377  12.677  50.010  1.00 11.68           H
ATOM   1003  HE1 HIS A 118       6.238  12.488  47.134  1.00 12.01           H
ATOM   1004  HE2 HIS A 118       8.361  13.744  47.911  1.00 12.02           H
ATOM   1693  CB  GLU A 204       0.166   9.088  43.640  1.00 12.74           H
ATOM   1694  CG  GLU A 204       0.886   8.274  43.880  1.00 12.07           C
ATOM   1695  CD  GLU A 204       2.153   8.417  43.057  1.00 12.52           C
ATOM   1696  OE1 GLU A 204       2.124   8.413  41.824  1.00 13.85           O
ATOM   1697  OE2 GLU A 204       3.272   8.522  43.746  1.00 13.00           O
ATOM   1698  HG2 GLU A 204       1.098   8.267  44.961  1.00 12.93           H
ATOM   1699  HG3 GLU A 204       0.389   7.322  43.600  1.00 12.61           H
ATOM   1971  CB  GLU A 238       1.810  13.597  45.643  1.00 11.00           H
ATOM   1972  CG  GLU A 238       1.068  12.778  45.514  1.00 13.23           C
ATOM   1973  CD  GLU A 238       1.701  11.426  45.832  1.00 13.88           C
ATOM   1974  OE1 GLU A 238       2.415  10.892  44.913  1.00 11.84           O
ATOM   1975  OE2 GLU A 238       1.506  10.913  46.963  1.00 15.72           O
ATOM   1976  HG2 GLU A 238       0.220  12.967  46.194  1.00 12.95           H
ATOM   1977  HG3 GLU A 238       0.723  12.811  44.464  1.00 12.55           H
ATOM   1992  CA  HIS A 241       3.975  11.481  40.788  1.00 10.81           H
ATOM   1995  CB  HIS A 241       3.958  10.979  41.770  1.00 10.45           C
ATOM   1996  CG  HIS A 241       5.229  11.146  42.535  1.00 10.35           C
ATOM   1997  ND1 HIS A 241       5.363  10.705  43.836  1.00 10.45           N
ATOM   1998  CD2 HIS A 241       6.426  11.704  42.139  1.00 10.74           C
ATOM   1999  CE1 HIS A 241       6.599  10.986  44.230  1.00 10.87           C
ATOM   2000  NE2 HIS A 241       7.271  11.606  43.236  1.00 10.60           N
ATOM   2001  HB2 HIS A 241       3.118  11.377  42.371  1.00 11.29           H
ATOM   2002  HB3 HIS A 241       3.727   9.905  41.609  1.00 11.62           H
ATOM   2003  HD2 HIS A 241       6.750  12.151  41.202  1.00 10.86           H
ATOM   2004  HE1 HIS A 241       7.005  10.770  45.224  1.00 11.12           H
ATOM   2005  HE2 HIS A 241       8.245  11.905  43.263  1.00 11.78           H
HETATM 5581 FE    FE A1003       5.098   9.912  48.333  1.00 12.18          Fe
HETATM 5582 FE    FE A1004       4.004   9.622  45.159  1.00 10.85          Fe
HETATM 5641  O   HOH A3041       2.731   8.532  46.678  1.00 14.44           O
HETATM 5642  H1  HOH A3041       3.167   8.115  47.478  1.00 13.41           H
HETATM 5643  H2  HOH A3041       2.179   9.323  46.977  1.00 13.83           H
HETATM 5653  O   HOH A3051       3.272  10.864  49.095  1.00 15.02           O
HETATM 5654  H1  HOH A3051       3.152  11.340  49.940  1.00 14.25           H
HETATM 5655  H2  HOH A3051       2.598  11.156  48.414  1.00 14.06           H
HETATM 6044  O   HOH A3440       4.616  10.589  46.700  1.00 13.71           O
```

```
REMARK Oxidised XFEL structure, O2- ligand, wx=3
REMARK /home/ulf/Cosmos/Kristoffer/RNR/XFEL_3.0/oxidised/o/wx/3
ATOM    721  CA  ASP A  84      26.756   6.180 -14.653  1.00 14.47           H
ATOM    724  CB  ASP A  84      27.006   5.682 -13.705  1.00 14.27           C
ATOM    725  CG  ASP A  84      27.743   4.371 -13.995  1.00 15.68           C
ATOM    726  OD1 ASP A  84      28.025   4.015 -15.160  1.00 15.67           O
ATOM    727  OD2 ASP A  84      28.078   3.695 -12.942  1.00 15.41           O
ATOM    728  HB2 ASP A  84      27.648   6.337 -13.086  1.00 16.12           H
ATOM    729  HB3 ASP A  84      26.092   5.473 -13.118  1.00 15.70           H
ATOM    971  CB  GLU A 115      23.978   1.543 -14.405  1.00 13.19           H
ATOM    972  CG  GLU A 115      24.545   0.865 -15.054  1.00 14.33           C
ATOM    973  CD  GLU A 115      26.043   1.013 -14.919  1.00 15.22           C
ATOM    974  OE1 GLU A 115      26.506   1.692 -13.937  1.00 15.48           O
ATOM    975  OE2 GLU A 115      26.720   0.440 -15.811  1.00 15.05           O
ATOM    976  HG2 GLU A 115      24.294  -0.182 -14.798  1.00 14.52           H
ATOM    977  HG3 GLU A 115      24.276   1.024 -16.116  1.00 14.87           H
ATOM    994  CA  HIS A 118      26.301   3.305  -9.191  1.00 12.81           H
ATOM    997  CB  HIS A 118      26.793   3.067 -10.150  1.00 12.12           C
ATOM    998  CG  HIS A 118      27.262   1.651 -10.179  1.00 14.35           C
ATOM    999  ND1 HIS A 118      27.921   1.119 -11.284  1.00 13.71           N
ATOM   1000  CD2 HIS A 118      27.120   0.643  -9.251  1.00 12.40           C
ATOM   1001  CE1 HIS A 118      28.197  -0.152 -11.026  1.00 13.98           C
ATOM   1002  NE2 HIS A 118      27.725  -0.469  -9.808  1.00 13.72           N
ATOM   1003  HB2 HIS A 118      26.081   3.247 -10.975  1.00 13.85           H
ATOM   1004  HB3 HIS A 118      27.634   3.764 -10.320  1.00 14.67           H
ATOM   1005  HD2 HIS A 118      26.651   0.620  -8.271  1.00 14.04           H
ATOM   1006  HE1 HIS A 118      28.728  -0.818 -11.713  1.00 14.98           H
ATOM   1007  HE2 HIS A 118      27.900  -1.344  -9.347  1.00 15.20           H
ATOM   1695  CB  GLU A 204      31.194  -0.269 -19.107  1.00 14.43           H
ATOM   1696  CG  GLU A 204      30.172   0.194 -19.034  1.00 15.78           C
ATOM   1697  CD  GLU A 204      29.100  -0.836 -18.709  1.00 16.83           C
ATOM   1698  OE1 GLU A 204      28.892  -1.797 -19.456  1.00 16.14           O
ATOM   1699  OE2 GLU A 204      28.416  -0.619 -17.599  1.00 14.71           O
ATOM   1700  HG2 GLU A 204      30.185   1.001 -18.282  1.00 16.00           H
ATOM   1701  HG3 GLU A 204      29.945   0.634 -20.027  1.00 16.18           H
ATOM   1984  CB  GLU A 238      32.728  -1.662 -14.169  1.00 14.92           H
ATOM   1985  CG  GLU A 238      32.832  -1.120 -15.133  1.00 15.50           C
ATOM   1986  CD  GLU A 238      31.596  -0.263 -15.404  1.00 16.72           C
ATOM   1987  OE1 GLU A 238      30.583  -0.857 -15.929  1.00 15.22           O
ATOM   1988  OE2 GLU A 238      31.643   0.961 -15.108  1.00 16.85           O
ATOM   1989  HG2 GLU A 238      33.722  -0.472 -15.050  1.00 17.01           H
ATOM   1990  HG3 GLU A 238      32.976  -1.868 -15.932  1.00 16.18           H
ATOM   2005  CA  HIS A 241      28.993  -4.776 -17.192  1.00 14.50           H
ATOM   2008  CB  HIS A 241      28.937  -3.703 -16.933  1.00 14.13           C
ATOM   2009  CG  HIS A 241      28.082  -3.456 -15.741  1.00 12.37           C
ATOM   2010  ND1 HIS A 241      27.954  -2.199 -15.162  1.00 13.54           N
ATOM   2011  CD2 HIS A 241      27.322  -4.346 -14.998  1.00 15.96           C
ATOM   2012  CE1 HIS A 241      27.149  -2.324 -14.106  1.00 16.06           C
ATOM   2013  NE2 HIS A 241      26.729  -3.608 -13.987  1.00 14.49           N
ATOM   2014  HB2 HIS A 241      29.959  -3.327 -16.737  1.00 15.11           H
ATOM   2015  HB3 HIS A 241      28.568  -3.149 -17.822  1.00 14.87           H
ATOM   2016  HD2 HIS A 241      27.162  -5.420 -15.097  1.00 15.65           H
ATOM   2017  HE1 HIS A 241      26.843  -1.514 -13.444  1.00 15.95           H
ATOM   2018  HE2 HIS A 241      26.078  -3.963 -13.292  1.00 16.46           H
HETATM 5632 FE    FE C   3      28.347   1.730 -13.178  1.00 16.40          Fe
HETATM 5633 FE    FE C   4      28.663  -0.259 -15.715  1.00 15.31          Fe
HETATM 5637  O   HOH D   4      28.884   0.153 -13.934  0.90 15.76           O
HETATM 5638  O   HOH D   5      30.445   2.283 -13.106  1.00 16.62           O
HETATM 5639  H1  HOH D   5      30.925   2.003 -12.302  1.00 16.17           H
HETATM 5640  H2  HOH D   5      30.908   1.788 -13.862  1.00 16.59           H
HETATM 5641  O   HOH D   6      29.254   1.822 -16.234  1.00 15.97           O
HETATM 5642  H1  HOH D   6      30.206   1.797 -15.934  1.00 16.14           H
HETATM 5643  H2  HOH D   6      28.806   2.642 -15.835  1.00 15.93           H
```

```
REMARK Oxidised MicroED structure, OH- ligand, wx=1
REMARK /home/ulf/Cosmos/Kristoffer/RNR/ED_2.0/oxidized_2.0/oh/wx/1
ATOM   7041  CA  ASP C  84       5.098 -10.126 -14.094  1.00 28.25           H
ATOM   7044  CB  ASP C  84       5.478 -10.540 -13.133  1.00 25.90           C
ATOM   7045  CG  ASP C  84       5.125  -9.707 -11.908  1.00 26.61           C
ATOM   7046  OD1 ASP C  84       4.684  -8.514 -11.967  1.00 35.51           O
ATOM   7047  OD2 ASP C  84       5.374 -10.188 -10.740  1.00 41.84           O
ATOM   7050  HB2 ASP C  84       6.580 -10.605 -13.174  1.00 29.58           H
ATOM   7051  HB3 ASP C  84       5.101 -11.570 -12.995  1.00 39.69           H
ATOM   7541  CB  GLU C 115       8.397  -6.254 -12.355  1.00 25.85           H
ATOM   7542  CG  GLU C 115       7.782  -5.444 -11.946  1.00 36.88           C
ATOM   7543  CD  GLU C 115       6.610  -5.952 -11.143  1.00 23.74           C
ATOM   7544  OE1 GLU C 115       6.583  -7.181 -10.823  1.00 32.89           O
ATOM   7545  OE2 GLU C 115       5.725  -5.111 -10.801  1.00 24.80           O
ATOM   7550  HG2 GLU C 115       8.404  -4.833 -11.269  1.00 25.13           H
ATOM   7551  HG3 GLU C 115       7.434  -4.784 -12.760  1.00 28.46           H
ATOM   7586  CA  HIS C 118       8.760 -11.593  -9.512  1.00 29.55           H
ATOM   7589  CB  HIS C 118       7.909 -10.896  -9.615  1.00 25.22           C
ATOM   7590  CG  HIS C 118       7.840  -9.910  -8.496  1.00 25.84           C
ATOM   7591  ND1 HIS C 118       6.882  -8.889  -8.443  1.00 29.68           N
ATOM   7592  CD2 HIS C 118       8.629  -9.793  -7.374  1.00 29.69           C
ATOM   7593  CE1 HIS C 118       7.103  -8.188  -7.327  1.00 21.88           C
ATOM   7594  NE2 HIS C 118       8.157  -8.703  -6.667  1.00 27.33           N
ATOM   7597  HB2 HIS C 118       8.011 -10.379 -10.584  1.00 24.70           H
ATOM   7598  HB3 HIS C 118       6.974 -11.481  -9.676  1.00 26.29           H
ATOM   7599  HD2 HIS C 118       9.479 -10.378  -7.016  1.00 22.03           H
ATOM   7600  HE1 HIS C 118       6.517  -7.337  -6.977  1.00 26.18           H
ATOM   7601  HE2 HIS C 118       8.470  -8.415  -5.749  1.00 28.47           H
ATOM   9041  CB  GLU C 204       0.306  -3.453  -9.964  1.00 31.22           H
ATOM   9042  CG  GLU C 204       1.064  -3.622 -10.752  1.00 35.59           C
ATOM   9043  CD  GLU C 204       2.306  -2.828 -10.436  1.00 40.34           C
ATOM   9044  OE1 GLU C 204       2.260  -1.629 -10.219  1.00 33.82           O
ATOM   9045  OE2 GLU C 204       3.453  -3.521 -10.428  1.00 31.59           O
ATOM   9050  HG2 GLU C 204       1.279  -4.697 -10.851  1.00 31.76           H
ATOM   9051  HG3 GLU C 204       0.635  -3.256 -11.706  1.00 31.18           H
ATOM   9661  CB  GLU C 238       1.947  -6.035  -5.561  1.00 32.34           H
ATOM   9662  CG  GLU C 238       1.253  -5.845  -6.421  1.00 31.80           C
ATOM   9663  CD  GLU C 238       2.006  -6.088  -7.720  1.00 38.94           C
ATOM   9664  OE1 GLU C 238       2.613  -5.072  -8.248  1.00 41.25           O
ATOM   9665  OE2 GLU C 238       2.074  -7.253  -8.201  1.00 48.70           O
ATOM   9670  HG2 GLU C 238       0.405  -6.545  -6.328  1.00 35.69           H
ATOM   9671  HG3 GLU C 238       0.898  -4.802  -6.351  1.00 36.29           H
ATOM   9702  CA  HIS C 241       4.172  -0.935  -7.207  1.00 23.30           H
ATOM   9705  CB  HIS C 241       4.152  -1.851  -7.797  1.00 26.24           C
ATOM   9706  CG  HIS C 241       5.411  -2.633  -7.687  1.00 25.51           C
ATOM   9707  ND1 HIS C 241       5.542  -3.903  -8.255  1.00 28.85           N
ATOM   9708  CD2 HIS C 241       6.614  -2.304  -7.096  1.00 29.10           C
ATOM   9709  CE1 HIS C 241       6.773  -4.324  -8.001  1.00 26.14           C
ATOM   9710  NE2 HIS C 241       7.460  -3.382  -7.304  1.00 22.43           N
ATOM   9713  HB2 HIS C 241       3.285  -2.455  -7.469  1.00 28.52           H
ATOM   9714  HB3 HIS C 241       3.962  -1.546  -8.844  1.00 27.00           H
ATOM   9715  HD2 HIS C 241       6.944  -1.411  -6.562  1.00 19.73           H
ATOM   9716  HE1 HIS C 241       7.200  -5.281  -8.315  1.00 29.11           H
ATOM   9717  HE2 HIS C 241       8.438  -3.430  -7.031  1.00 31.70           H
HETATM11365 FE    FE A   1       5.360  -8.291  -9.759  1.00 25.29          Fe
HETATM11367 FE    FE A   3       4.199  -5.011  -9.541  1.00 25.12          Fe
HETATM11463  O   HOH S 109       4.814  -6.778  -8.650  1.00 26.67           O
HETATM11464  H1  HOH S 109       4.109  -7.013  -8.007  1.00 25.27           H
HETATM11515  O   HOH S 191       3.733  -9.181  -8.808  1.00 42.37           O
HETATM11516  H1  HOH S 191       2.948  -8.603  -8.534  1.00 39.21           H
HETATM11517  H2  HOH S 191       3.503 -10.127  -8.909  1.00 28.29           H
HETATM11646  O   HOH S 434       3.229  -6.414 -10.956  1.00 36.87           O
HETATM11647  H1  HOH S 434       2.375  -6.804 -10.670  1.00 34.62           H
HETATM11648  H2  HOH S 434       3.657  -7.043 -11.598  1.00 35.45           H
```

```
REMARK Reduced SCX structure, no ligand, wx=1
REMARK /home/ulf/Cosmos/Kristoffer/RNR/1xik/wx/1
ATOM    718  CA  ASP A  84       5.161   5.661  50.453  1.00 17.60           H
ATOM    721  CB  ASP A  84       5.601   6.565  50.910  1.00 20.51           C
ATOM    722  CG  ASP A  84       5.204   7.810  50.110  1.00 20.26           C
ATOM    723  OD1 ASP A  84       5.423   7.750  48.822  1.00 22.80           O
ATOM    724  OD2 ASP A  84       4.754   8.832  50.661  1.00 19.47           O
ATOM    725  HB2 ASP A  84       5.266   6.658  51.958  1.00 20.91           H
ATOM    726  HB3 ASP A  84       6.701   6.452  50.882  1.00 20.41           H
ATOM    968  CB  GLU A 115       7.869   6.683  46.434  1.00 17.72           H
ATOM    969  CG  GLU A 115       7.290   6.883  45.509  1.00 17.21           C
ATOM    970  CD  GLU A 115       6.129   7.856  45.642  1.00 20.46           C
ATOM    971  OE1 GLU A 115       6.245   8.921  46.329  1.00 20.96           O
ATOM    972  OE2 GLU A 115       5.109   7.602  44.922  1.00 20.83           O
ATOM    973  HG2 GLU A 115       7.987   7.316  44.765  1.00 19.01           H
ATOM    974  HG3 GLU A 115       6.919   5.929  45.096  1.00 18.94           H
ATOM    991  CA  HIS A 118       8.519  10.344  51.246  1.00 18.59           H
ATOM    994  CB  HIS A 118       7.645  10.109  50.628  1.00 18.30           C
ATOM    995  CG  HIS A 118       7.594  10.993  49.440  1.00 18.47           C
ATOM    996  ND1 HIS A 118       6.673  10.809  48.425  1.00 17.91           N
ATOM    997  CD2 HIS A 118       8.366  12.093  49.140  1.00 18.27           C
ATOM    998  CE1 HIS A 118       6.883  11.759  47.525  1.00 19.22           C
ATOM    999  NE2 HIS A 118       7.905  12.545  47.923  1.00 20.52           N
ATOM   1000  HB2 HIS A 118       7.716   9.054  50.312  1.00 19.60           H
ATOM   1001  HB3 HIS A 118       6.723  10.196  51.233  1.00 19.65           H
ATOM   1002  HD2 HIS A 118       9.196  12.571  49.659  1.00 19.98           H
ATOM   1003  HE1 HIS A 118       6.338  11.891  46.590  1.00 20.34           H
ATOM   1004  HE2 HIS A 118       8.117  13.437  47.519  1.00 19.84           H
ATOM   1693  CB  GLU A 204       0.061   8.468  42.092  1.00 21.65           H
ATOM   1694  CG  GLU A 204       0.816   7.675  42.000  1.00 23.42           C
ATOM   1695  CD  GLU A 204       2.073   7.880  42.842  1.00 22.35           C
ATOM   1696  OE1 GLU A 204       2.234   9.054  43.394  1.00 21.15           O
ATOM   1697  OE2 GLU A 204       2.904   6.967  42.935  1.00 22.80           O
ATOM   1698  HG2 GLU A 204       0.375   6.691  42.237  1.00 22.86           H
ATOM   1699  HG3 GLU A 204       1.126   7.635  40.934  1.00 21.35           H
ATOM   1972  CB  GLU A 238       2.164  12.863  45.511  1.00 21.03           H
ATOM   1973  CG  GLU A 238       2.555  12.574  46.504  1.00 24.16           C
ATOM   1974  CD  GLU A 238       3.341  11.276  46.493  1.00 26.35           C
ATOM   1975  OE1 GLU A 238       3.449  10.635  45.424  1.00 23.36           O
ATOM   1976  OE2 GLU A 238       3.866  10.918  47.610  1.00 26.19           O
ATOM   1977  HG2 GLU A 238       3.192  13.381  46.907  1.00 22.06           H
ATOM   1978  HG3 GLU A 238       1.705  12.478  47.209  1.00 24.19           H
ATOM   1993  CA  HIS A 241       3.924  10.918  40.182  1.00 19.29           H
ATOM   1996  CB  HIS A 241       3.922  10.390  41.143  1.00 18.71           C
ATOM   1997  CG  HIS A 241       5.224  10.580  41.851  1.00 17.24           C
ATOM   1998  ND1 HIS A 241       5.390  10.183  43.176  1.00 19.56           N
ATOM   1999  CD2 HIS A 241       6.412  11.124  41.406  1.00 20.33           C
ATOM   2000  CE1 HIS A 241       6.643  10.480  43.523  1.00 19.14           C
ATOM   2001  NE2 HIS A 241       7.290  11.052  42.473  1.00 18.34           N
ATOM   2002  HB2 HIS A 241       3.076  10.749  41.758  1.00 19.72           H
ATOM   2003  HB3 HIS A 241       3.748   9.313  40.950  1.00 19.85           H
ATOM   2004  HD2 HIS A 241       6.696  11.557  40.447  1.00 18.43           H
ATOM   2005  HE1 HIS A 241       7.090  10.265  44.495  1.00 19.28           H
ATOM   2006  HE2 HIS A 241       8.273  11.315  42.452  1.00 19.63           H
HETATM 5610 FE    FE A 376       5.142   9.530  48.014  1.00 17.00          Fe
HETATM 5611 FE    FE A 377       4.041   9.131  44.269  1.00 18.63          Fe
```

```
REMARK Reduced XFEL structure, H2O, wx=3
REMARK /home/ulf/Cosmos/Kristoffer/RNR/XFEL_3.0/reduced/hoh/wx/3
ATOM   3566  CA  ASP B  84       5.160  -9.907 -14.255  1.00 15.86           H
ATOM   3569  CB  ASP B  84       5.565 -10.279 -13.294  1.00 18.97           C
ATOM   3570  CG  ASP B  84       5.111  -9.369 -12.125  1.00 19.17           C
ATOM   3571  OD1 ASP B  84       4.450  -8.325 -12.329  1.00 21.56           O
ATOM   3572  OD2 ASP B  84       5.454  -9.770 -10.944  1.00 19.78           O
ATOM   3573  HB2 ASP B  84       5.212 -11.310 -13.104  1.00 18.62           H
ATOM   3574  HB3 ASP B  84       6.668 -10.310 -13.339  1.00 17.66           H
ATOM   3829  CB  GLU B 115       8.369  -6.184 -12.451  1.00 16.02           H
ATOM   3830  CG  GLU B 115       7.724  -5.357 -12.106  1.00 15.33           C
ATOM   3831  CD  GLU B 115       6.534  -5.702 -11.234  1.00 18.68           C
ATOM   3832  OE1 GLU B 115       6.540  -6.799 -10.574  1.00 18.99           O
ATOM   3833  OE2 GLU B 115       5.645  -4.818 -11.162  1.00 15.23           O
ATOM   3834  HG2 GLU B 115       8.360  -4.670 -11.518  1.00 16.12           H
ATOM   3835  HG3 GLU B 115       7.361  -4.792 -12.985  1.00 16.49           H
ATOM   3852  CA  HIS B 118       8.841 -11.438  -9.501  1.00 14.96           H
ATOM   3855  CB  HIS B 118       7.970 -10.767  -9.601  1.00 14.46           C
ATOM   3856  CG  HIS B 118       7.926  -9.778  -8.500  1.00 14.14           C
ATOM   3857  ND1 HIS B 118       6.963  -8.772  -8.459  1.00 16.99           N
ATOM   3858  CD2 HIS B 118       8.744  -9.630  -7.407  1.00 15.52           C
ATOM   3859  CE1 HIS B 118       7.192  -8.051  -7.369  1.00 18.60           C
ATOM   3860  NE2 HIS B 118       8.268  -8.535  -6.711  1.00 16.38           N
ATOM   3861  HB2 HIS B 118       8.023 -10.256 -10.579  1.00 15.83           H
ATOM   3862  HB3 HIS B 118       7.044 -11.369  -9.638  1.00 16.09           H
ATOM   3863  HD2 HIS B 118       9.619 -10.184  -7.076  1.00 17.50           H
ATOM   3864  HE1 HIS B 118       6.589  -7.199  -7.047  1.00 17.14           H
ATOM   3865  HE2 HIS B 118       8.558  -8.249  -5.794  1.00 17.06           H
ATOM   4566  CB  GLU B 204       0.303  -2.293  -9.673  1.00 18.10           H
ATOM   4567  CG  GLU B 204       1.141  -2.197 -10.389  1.00 19.16           C
ATOM   4568  CD  GLU B 204       2.293  -3.142 -10.115  1.00 17.03           C
ATOM   4569  OE1 GLU B 204       2.251  -3.950  -9.131  1.00 19.99           O
ATOM   4570  OE2 GLU B 204       3.305  -3.084 -10.874  1.00 19.84           O
ATOM   4571  HG2 GLU B 204       0.774  -2.380 -11.417  1.00 19.36           H
ATOM   4572  HG3 GLU B 204       1.534  -1.161 -10.363  1.00 18.06           H
ATOM   4874  CB  GLU B 238       2.509  -6.270  -5.980  1.00 18.72           H
ATOM   4875  CG  GLU B 238       2.874  -7.202  -6.490  1.00 20.50           C
ATOM   4876  CD  GLU B 238       3.722  -7.116  -7.759  1.00 22.20           C
ATOM   4877  OE1 GLU B 238       4.308  -6.045  -8.075  1.00 21.05           O
ATOM   4878  OE2 GLU B 238       3.907  -8.212  -8.399  1.00 20.22           O
ATOM   4879  HG2 GLU B 238       3.459  -7.793  -5.764  1.00 19.39           H
ATOM   4880  HG3 GLU B 238       1.986  -7.822  -6.715  1.00 22.06           H
ATOM   4895  CA  HIS B 241       4.325  -0.663  -7.233  1.00 15.68           H
ATOM   4898  CB  HIS B 241       4.298  -1.540  -7.895  1.00 17.23           C
ATOM   4899  CG  HIS B 241       5.558  -2.328  -7.824  1.00 15.68           C
ATOM   4900  ND1 HIS B 241       5.643  -3.565  -8.439  1.00 15.65           N
ATOM   4901  CD2 HIS B 241       6.764  -2.061  -7.198  1.00 18.01           C
ATOM   4902  CE1 HIS B 241       6.855  -4.035  -8.208  1.00 16.46           C
ATOM   4903  NE2 HIS B 241       7.572  -3.159  -7.447  1.00 16.59           N
ATOM   4904  HB2 HIS B 241       3.437  -2.179  -7.624  1.00 16.40           H
ATOM   4905  HB3 HIS B 241       4.118  -1.195  -8.931  1.00 15.62           H
ATOM   4906  HD2 HIS B 241       7.102  -1.220  -6.597  1.00 18.37           H
ATOM   4907  HE1 HIS B 241       7.244  -4.992  -8.572  1.00 17.32           H
ATOM   4908  HE2 HIS B 241       8.533  -3.277  -7.138  1.00 17.72           H
HETATM 5721 FE   FE2 C   1       5.426  -8.204  -9.700  1.00 17.12          Fe
HETATM 5722 FE   FE2 C   2       4.246  -4.706  -9.658  1.00 16.82          Fe
HETATM 5725  O   HOH D   1       3.187  -6.239 -10.869  1.00 20.76           O
HETATM 5726  H1  HOH D   1       3.595  -6.911 -11.491  1.00 20.00           H
HETATM 5727  H2  HOH D   1       2.252  -6.478 -10.718  1.00 21.58           H
```

```
REMARK Reduced MicroED structure, H2O, wx=1
REMARK /home/ulf/Cosmos/Kristoffer/RNR/ED_2.0/reduced/hoh/wx/1
ATOM   7076  CA  ASP C  84       5.371  -9.891 -14.263  1.00 27.28           H
ATOM   7079  CB  ASP C  84       5.766 -10.324 -13.321  1.00 28.70           C
ATOM   7080  CG  ASP C  84       5.251  -9.482 -12.128  1.00 19.83           C
ATOM   7081  OD1 ASP C  84       4.771  -8.319 -12.311  1.00 41.93           O
ATOM   7082  OD2 ASP C  84       5.438  -9.938 -10.946  1.00 29.62           O
ATOM   7085  HB2 ASP C  84       6.873 -10.298 -13.323  1.00 21.93           H
ATOM   7086  HB3 ASP C  84       5.456 -11.379 -13.210  1.00 23.89           H
ATOM   7565  CB  GLU C 115       8.580  -6.189 -12.587  1.00 35.68           H
ATOM   7566  CG  GLU C 115       7.984  -5.332 -12.249  1.00 23.81           C
ATOM   7567  CD  GLU C 115       6.823  -5.667 -11.320  1.00 25.59           C
ATOM   7568  OE1 GLU C 115       6.864  -6.761 -10.648  1.00 22.46           O
ATOM   7569  OE2 GLU C 115       5.944  -4.775 -11.176  1.00 15.03           O
ATOM   7574  HG2 GLU C 115       8.655  -4.652 -11.688  1.00 24.63           H
ATOM   7575  HG3 GLU C 115       7.619  -4.774 -13.132  1.00 21.95           H
ATOM   7610  CA  HIS C 118       9.035 -11.434  -9.713  1.00 22.07           H
ATOM   7613  CB  HIS C 118       8.161 -10.755  -9.840  1.00 19.53           C
ATOM   7614  CG  HIS C 118       8.050  -9.769  -8.720  1.00 23.42           C
ATOM   7615  ND1 HIS C 118       7.103  -8.754  -8.710  1.00 28.63           N
ATOM   7616  CD2 HIS C 118       8.820  -9.658  -7.584  1.00 23.20           C
ATOM   7617  CE1 HIS C 118       7.294  -8.046  -7.608  1.00 19.91           C
ATOM   7618  NE2 HIS C 118       8.327  -8.559  -6.902  1.00 21.12           N
ATOM   7621  HB2 HIS C 118       8.264 -10.224 -10.800  1.00 24.58           H
ATOM   7622  HB3 HIS C 118       7.244 -11.369  -9.939  1.00 20.96           H
ATOM   7623  HD2 HIS C 118       9.676 -10.231  -7.229  1.00 22.48           H
ATOM   7624  HE1 HIS C 118       6.684  -7.196  -7.287  1.00 22.39           H
ATOM   7625  HE2 HIS C 118       8.635  -8.226  -6.002  1.00 27.41           H
ATOM   9062  CB  GLU C 204       0.530  -2.257  -9.724  1.00 25.64           H
ATOM   9063  CG  GLU C 204       1.340  -2.196 -10.472  1.00 28.21           C
ATOM   9064  CD  GLU C 204       2.451  -3.177 -10.159  1.00 18.88           C
ATOM   9065  OE1 GLU C 204       2.335  -4.023  -9.224  1.00 36.55           O
ATOM   9066  OE2 GLU C 204       3.525  -3.112 -10.857  1.00 27.13           O
ATOM   9071  HG2 GLU C 204       0.927  -2.431 -11.472  1.00 32.31           H
ATOM   9072  HG3 GLU C 204       1.745  -1.167 -10.527  1.00 20.97           H
ATOM   9614  CB  GLU C 238       2.614  -6.188  -6.071  1.00 26.47           H
ATOM   9615  CG  GLU C 238       2.996  -7.130  -6.547  1.00 29.37           C
ATOM   9616  CD  GLU C 238       3.854  -6.952  -7.796  1.00 32.39           C
ATOM   9617  OE1 GLU C 238       4.275  -5.807  -8.086  1.00 31.89           O
ATOM   9618  OE2 GLU C 238       4.154  -8.009  -8.466  1.00 58.81           O
ATOM   9623  HG2 GLU C 238       3.583  -7.686  -5.792  1.00 30.91           H
ATOM   9624  HG3 GLU C 238       2.138  -7.783  -6.800  1.00 30.05           H
ATOM   9655  CA  HIS C 241       4.529  -0.631  -7.330  1.00 26.35           H
ATOM   9658  CB  HIS C 241       4.509  -1.525  -7.967  1.00 13.01           C
ATOM   9659  CG  HIS C 241       5.767  -2.347  -7.907  1.00 21.95           C
ATOM   9660  ND1 HIS C 241       5.800  -3.604  -8.496  1.00 18.59           N
ATOM   9661  CD2 HIS C 241       7.025  -2.060  -7.413  1.00 16.44           C
ATOM   9662  CE1 HIS C 241       7.032  -4.069  -8.359  1.00 27.77           C
ATOM   9663  NE2 HIS C 241       7.807  -3.164  -7.695  1.00 16.26           N
ATOM   9666  HB2 HIS C 241       3.645  -2.146  -7.662  1.00 23.61           H
ATOM   9667  HB3 HIS C 241       4.322  -1.201  -9.010  1.00 19.10           H
ATOM   9668  HD2 HIS C 241       7.413  -1.201  -6.864  1.00 17.95           H
ATOM   9669  HE1 HIS C 241       7.384  -5.024  -8.754  1.00 19.96           H
ATOM   9670  HE2 HIS C 241       8.788  -3.286  -7.441  1.00 22.31           H
HETATM11310 FE    FE A   1       5.549  -8.103  -9.951  1.00 32.52          Fe
HETATM11312 FE    FE A   3       4.425  -4.721  -9.797  1.00 22.51          Fe
HETATM11425  O   HOH S 130       3.389  -6.350 -10.842  1.00 24.82           O
HETATM11426  H1  HOH S 130       3.765  -7.018 -11.490  1.00 30.05           H
HETATM11427  H2  HOH S 130       2.588  -6.732 -10.426  1.00 27.11           H
```
